# Natural Products as Hepatoprotective Agents—A Comprehensive Review of Clinical Trials

**DOI:** 10.3390/plants13141985

**Published:** 2024-07-20

**Authors:** Piotr Służały, Paweł Paśko, Agnieszka Galanty

**Affiliations:** 1 Department of Pharmacognosy, Jagiellonian University Medical College, Medyczna 9, 30-688 Cracow, Poland; piotr_s-95@o2.pl; 2 Department of Food Chemistry and Nutrition, Jagiellonian University Medical College, Medyczna 9, 30-688 Cracow, Poland; p.pasko@uj.edu.pl

**Keywords:** liver, herbal medication, hepatoprotective, clinical trials

## Abstract

The hepatoprotective effects of natural products have been a significant focus in recent decades due to the growing demand for the help in the treatment of hepatic impairments. This review specifically delves into the findings of clinical trials involving 13 selected natural products, namely plants and their derived compounds (e.g., artichoke, berberine, and turmeric), algae (e.g., spirulina), probiotics, and other products like phospholipids and vitamin D. A literature search was performed in the Scopus database, PubMed, and Google Scholar, covering all articles found up to June 2024. Artichoke, berberine, chlorella, chicory, green tea, probiotics, phospholipids, schisandra, silymarin, spirulina, and vitamin D caused a decrease in liver enzymes, while for cinnamon and turmeric such an effect was either not observed or not convincing. The presented results indicate that some natural products might satisfactorily improve hepatic outcomes in NAFLD, NASH, and other liver disorders; however, further studies and metanalyses are needed to clearly demonstrate their effectiveness.

## 1. Introduction

The liver is one of the most important organs of the human body, with a crucial role in physiological processes such as bile secretion, synthesis of vital proteins like albumins and fibrinogen, and also storage and metabolism. The latter role is especially critical in terms of drug metabolism within the body, impacting the safety, pharmacological effectiveness, and potential hepatotoxicity of medications. Despite all of those integral functions, hepatic impairments continue to become a major health concern worldwide [1]. Damage to hepatocytes, connective tissue, or the liver as an organ itself may be induced by autoimmune diseases (primary biliary, cirrhosis, or immune hepatitis) and biological factors (viruses, bacteria, and other organisms). The overuse of chemicals (e.g., thioacetamide, carbon tetrachloride, and ethanol) or drugs (e.g., acetaminophen, antituberculars, antiepileptics, and cytostatics) could also affect hepatic functioning [2]. Chronic liver diseases have become a main cause of mortality and morbidity over the last decade, with as many as two million annual deaths globally [3]. The common reasons for chronic liver disease are non-alcoholic fatty liver disease (NAFLD), hepatitis B and C, cirrhosis, and hepatocellular carcinoma which, as complications, contribute to liver-related mortality [4]. Metabolic risk factors for NAFLD include features of the metabolic syndrome that indicate increased cardiovascular risk. According to Bedogni et al. [5], 91% of those affected by NAFLD are overweight or obese [5]. Additionally, 55% of individuals with NAFLD also suffer from impaired glucose regulation or type 2 diabetes mellitus (T2DM), as noted by Ballestri et al. [6]. Other contributing factors may include hypertension, hyperlipidemia, family history, and the use of certain drugs such as tamoxifen, methotrexate, and diltiazem.

NAFLD is a public health problem with a high prevalence, directly impacting around 11–16% of the world population. This pathological syndrome is characterized by excessive build-up of lipids in hepatocytes. The mechanism of this state is an imbalance between the input and output of free fatty acid metabolism in liver tissue [7]. Pathogenesis of NAFLD was recently linked to metabolic disturbances and changes in the glucose–insulin relation. Therefore, the name of NAFLD was re-evaluated to the newer name of metabolic (impairment)-associated fatty liver disease (MAFLD).

Despite great discoveries in modern pharmacology, no effective medication may cure completely or show an enormous improvement in hepatic functioning and the regeneration of hepatocytes [8]. The pharmacological treatment options for NAFLD include insulin sensitizers (metformin, rosiglitazone, and semaglutide), lipid-lowering drugs (clofibrate, gemfibrozil, and atorvastatin), angiotensin receptor blockers (telmisartan and candesartan), pentoxifylline, ursodeoxycholic acid, and antioxidants, but also herbal medicines [9]. A well-established therapy includes weight loss and dietary intervention that may significantly increase the effectiveness of medication treatments [10]. Recently, the treatment methods recommended by gastroenterology guidelines include lifestyle intervention as a holistic approach, drug treatment, and surgical procedures [11]. However, some natural products, both as drugs and as diet elements and dietary supplements, have been widely used as supports in liver functioning or in the prevention of liver diseases’ development. The use of these natural products is based on their traditional uses, but the effectiveness of some of them have been examined in animal and human studies. However, the question arises of which natural product is worth choosing, in terms of its effectiveness in liver disorders. Thus, the aim of this review is to collect the data on the so far performed clinical trials of natural products, including plants, their derived compounds, and algae, microorganisms, or vitamins, and to critically compare their use in NAFLD and some other liver diseases.

Search strategy

A literature search was conducted in the Scopus database, PubMed, and Google Scholar, covering all articles found up to July 2024. Initially, the search term “liver and clinical trials and plants” was used, and then the term “liver and clinical trials and silymarin” was used in combination with the following: chicory, turmeric, artichoke, schisandra, berberine, vitamin D, green tea, spirulina, and probiotics. For example, “liver and clinical trials and silymarin” found 1708 articles. An additional criterion was introduced to include only English language results. After checking the titles and abstracts, 456 items were excluded due to being trials other than clinical and/or human trials or reviews. Only 289 were included with relevance to this review. Of the remaining 202 articles, a total of 84 were used for this review (Figure S1).

For the purposes of this review, the details of the studies performed are gathered in the appropriate tables in the Supplementary Material. To facilitate the understanding of the presented effects of the examined substances on liver functioning, different outcome measures are briefly characterized below.

Liver functioning outcome measurements

Determination of the levels of alanine aminotransferase (ALT), aspartate aminotransferase (AST), alkaline phosphatase (ALP), or gamma-glutamyl transferase (GGT) can assist in identifying specific areas of the liver that may be experiencing damage and, based on their pattern of elevation, can aid in proper diagnosis. Elevations in ALT and AST in comparison to the elevations in ALP and bilirubin indicate hepatocellular disease [3]. Conversely, an elevation in ALP and bilirubin in comparison to ALT and AST would indicate a cholestatic pattern [2]. The activity of these enzymes is presented in the manuscript as a percentage difference between baseline parameters and post-intervention results in both the intervention and placebo groups.

The fatty liver index (FLI) is a comprehensive algorithm that incorporates body mass index (BMI), waist circumference (WC), GGT, and triglyceride (TG) levels. It has been shown to be an effective tool in predicting the presence of NAFLD, due to its strong correlation with both imaging and histological indicators of the disease [4].

The lipid accumulation product (LAP) is an index that combines WC and TG levels to assess lipid accumulation. Higher LAP results indicate a more severe liver condition [12].

The hepatic vein waveform typically shows a triphasic pattern, characterized by retrograde flow during atrial contraction and two antegrade pulses during both ventricular systole and ventricular diastole [7].

A liver ultrasound is a non-invasive and straightforward diagnostic tool used to assess for a variety of liver conditions, such as cirrhosis, fatty liver disease, cancer, and other abnormalities: adipose accumulations within the hepatic organ, indicative of potential steatosis, fibrous tissue development or sclerosis within the hepatic organ, potentially signaling cirrhotic changes, abnormal growths within the liver, possibly suggestive of malignancies or infectious processes, or manifestations of hepatitis, inclusive of hepatic inflammation [9]. These include (i) the hepatorenal index, a semiquantitative ultrasonographic examination to estimate liver steatosis, calculated by dividing the mean brightness of the liver by the mean brightness of the kidneys; (ii) doppler flow, comprising the hemodynamic parameters of the portal vein, including flow pressure, flow volume rate, and wall shear stress (WSS); (iii) the liver–spleen (L/S) attenuation ratio, of significant value in the diagnostic assessment of hepatosteatosis due to its inherent benefits, including its ability to be measured within the same cross-sectional imaging study and its relative insensitivity to the presence of most systemic diseases; (iv) the diameter of the portal vein, which typically falls within a range of approximately 7–15 mm; and (v) the liver fatty content, where ultrasound technology is utilized to measure the attenuation of ultrasound waves in the liver for the purpose of assessing hepatic fat content [11].

## 2. Plants and Compounds Derived from Plants

### 2.1. Artichoke

Artichoke (*Cynara cardunculus* L.) is a plant widely consumed in the Mediterranean diet. Pharmacological effects are related to the presence of antioxidants, such as cynarin, chlorogenic acid, caffeic acid and its derivatives, and the volatile sesquiterpene and flavonoids, including the glycosides of luteolin [13]. The hepatoprotective mechanism is connected to cholesterol reduction, as artichoke is well known for decreasing the reactive oxygen species level, lipid peroxidation, protein oxidation, the oxidation of LDL-cholesterol, and causing an increase in glutathione peroxidase activity [14,15].

A total of eight randomized clinical trials were conducted to evaluate the impact of artichoke on liver enzymes in patients with NAFLD (*n* = 299) or hypercholesterolemia (*n* = 267). The research took place in various countries including Iran, Italy, France, and Germany. In the case of patients with NAFLD, the artichoke extract was administered in a range, from 300 to 2700 mg/day, over a period of 2–4 months, and in one case the artichoke was combined with a bergamot polyphenolic fraction (Table S1). The results indicated a significant decrease in liver parameters (ALT and AST), with a clear correlation between the dosage used and the observed effectiveness, demonstrating that the higher doses of the artichoke extract were more effective in inducing hepatic enzyme changes compared to the lower ones. Moreover, liver ultrasonography showed a significant improvement in terms of fat accumulation in the liver in two artichoke groups [16]. Doppler sonography ultimately revealed that analogous advantageous effects were unveiled due to the increase in hepatic vein flow, the enlargement of the liver size, and the reduction in portal vein diameter [17]. The beneficial effects of the artichoke combined with the bergamot polyphenolic fraction were manifested as an improvement in the hepatorenal index and liver brightness [18].

For patients with hypercholesterolemia, artichoke extract alone, but also other plant extracts and vitamins included in the combination, was administered in a range, from 20 to 450 mg/day, for a period of 4 to 8 weeks. However, the results of these studies are inconclusive, as two trials revealed significant changes observed in liver enzymes [14,19], while the other two showed the opposite effects [15,20]. Interestingly, in one of the studies, despite no observed effect on liver enzymes, the NAFLD indexes (lipid accumulation product and fatty liver index) were significantly improved [20]. The highest daily dose used in the studies was 2700 mg of the artichoke extract [21].

### 2.2. Berberine

Berberine is a quaternary isoquinoline alkaloid found in an ancient Chinese herb, *Coptis chinensis* Franch., and *Berberis aristata* DC., which has been used to treat diabetes for many years [22]. The efficacy of berberine in liver-related issues is connected to its cholesterol-lowering effect by the induction of the expression of LDL-cholesterol in hepatocytes’ post-transcription mRNA stabilization to encoding receptors [23]. Berberine has been proven to significantly reduce hepatic fat content in rats with NAFLD by decreasing methylation of the MTTP (microsomal triglyceride transfer protein) promoter and mitigating the hepatic steatosis caused by a high-fat dietary regimen in rodent models [24].

In total, five randomized clinical trials (Table S2) conducted in China, the USA, and Iran evaluated the impact of berberine on liver enzymes in patients with NAFLD and NASH [22,23,24,25,26]. A total of 478 patients aged 40–54 with liver impairment participated in the studies, which included assessments of ALT, AST, and GGT levels. Berberine dosages ranged from 1 to 6.25 g/day, with intervention periods lasting from 6 to 18 weeks. The results demonstrated a significant improvement in liver parameters, with a clear dose–response relationship observed in one study comparing 1 and 2 g doses. Higher doses of berberine were associated with greater reductions in hepatic enzyme levels. Additionally, a study by Yan et al. [23] showed a significant reduction in liver fat, which was assessed by a proton magnetic resonance spectroscopy, in the berberine group compared to the placebo and reference groups [23]. A greater reduction in liver fat content, measured by spectroscopy, was also confirmed by Harrison et al., with a dose-dependent effect noted for berberine compared to the placebo group [25].

### 2.3. Chicory

Chicory (*Cichorium intybus* L.) has been traditionally used for the treatment of liver diseases for thousands of years. It has antioxidant properties which are likely to be due to the presence of polyphenolic compounds [27,28]. Many trials have suggested that inulin, which is 40% present in chicory root extract, is effective in enhancing the expression of iron genes, enzymes, and ferritin in enterocytes, which corresponds to a reduction in serum ferritin and a change in liver enzyme levels [29].

A total of 374 individuals with NAFLD or cirrhosis or hepatobiliary issues were studied (Table S3) to evaluate their liver parameters following the consumption of chicory [27,28,29,30,31,32,33,34,35]. One study from India and eight studies from Iran were included in the analysis. Six studies focused solely on chicory in various forms, such as a water–plant, chicory leaf, a powder extract of the root, and powdered seeds. The interventions involved different doses of the mentioned extracts over a period ranging from 4 weeks to 6 months. Only one of the nine studies did not show significant improvements in liver parameters, with 61 NAFLD patients, where the intervention group received the lowest dose of chicory at 500 mg/day of powdered chicory root, while both the intervention and placebo groups were advised to make lifestyle modifications to meet dietary requirements. The results did not demonstrate significant changes between the intervention and placebo groups [30]. Overall, improvements in liver parameters may be attributed to lifestyle changes and dietary adjustments.

Outstanding results were noted in a study by Ghaffari et al., where the intervention included turmeric and chicory, alone or in combination. The highest impact in ALT serum levels was seen in the turmeric group at −21.1%, then in the group taking a combination of both substances at −17.5%, and the lowest was seen in the chicory group at −14.0%, vs. the placebo group at −3.5% [31]. A significant reduction in liver parameters was noted by Huseini et al., in which chicory was one of eight herbal ingredients (Liv-52). Unfortunately, it cannot be clearly stated if the effect was related to chicory or the synergistic impact of the other ingredients [32]. Another study was conducted by Parveen et al. on 30 subjects with hepatobiliary disease where the intervention included the combination of chicory and *Solanum nigrum*, but with no placebo group. The results showed significant improvements in liver parameters [28]. Significant decreases in the grade of sonographic examinations were assessed in a study by Sheybani et al. compared to the placebo group [33]. Marzaban et al. found a similar outcome in terms of the grade of NAFLD [27].

### 2.4. Cinnamon

Cinnamon is widely used as a spice in food preparation and herbal remedies for some diseases and inflammation. This herbal product is found in candy, toothpaste, and perfumes. Cinnamaldehyde is the main compound of cinnamon and can regulate immunological function via anti- and pro-inflammatory gene expression. This has been confirmed in many in-vitro and in-vivo studies. Traditionally, it is used as antioxidant and for the regulation of lipid profiles and glucose [36].

A sole, double-blind RCT study describing the impact of cinnamon (*Cinnamomum verum* J. Presl) on the liver was performed by Askari et al. [36] on 45 patients diagnosed with NAFLD in Iran. The researchers aimed to investigate a potential association between the daily consumption of 1.5 g of concentrated cinnamon extract (*n* = 23, 10 F, 13 M mean age 44.8, and BMI 29.9) over a 12-week period compared to a placebo group (*n* = 22, 13 F, 9 M mean age 45.4, and BMI 30.3). The trial focused on assessing the levels of liver enzymes such as ALT, AST, and GGT. The results showed a notable improvement in all liver parameters in the group that received the intervention. The ALT levels decreased by 37.25% compared to 2.07% in the placebo group, AST levels decreased by 36.73% compared to 2.77% in the placebo group, and GGT levels decreased by 32.47% compared to 3.46% in the placebo group [36].

### 2.5. Curcumin

Curcumin is a phenolic compound found in the roots of turmeric (*Curcuma longa* L.), a plant widely grown in India, China, and Indonesia [37]. Curcumin has been intensively studied for its anti-inflammatory, lipid-regulation, anti-arthritic, antioxidant, immunomodulatory, antidepressant, and anti-cancer potential. However, the health benefits of the compound in humans are problematic due to its low bioavailability [38].

A total of 575 patients with NAFLD and/or liver cirrhosis were included in the analysis. The studies (Table S4) were conducted in Italy and Iran [37,38,39,40,41,42,43,44]. Most of the studies were conducted using curcumin. Only one study included turmeric at a daily dosage of 2 g, with the remaining studies focusing on the standardization of curcumin. The most recent formulations of curcumin that have been studied include synacurcumin (80 mg), an amorphous dispersion curcumin formulation (equivalent to 70 mg of curcumin), 800 mg of phytosomal curcumin, and a combination of 500 mg of curcuminoids with 5 mg of piperine, which is used to enhance the bioavailability of curcuminoids. The majority of the studies (seven out of eight) reported improvements in liver parameters. The follow-up period of the studies ranged from 4 weeks to 6 months.

Hepatic fibrosis was assessed as a parameter by Saadati et al., with a significant reduction in the curcumin group only [38]. Ultrasonographic examination in another study demonstrated an improvement of the reduction in liver fat content in 78.9% of participants in the curcumin group, compared to only 27.5% in the placebo group. No instances of increased liver fat content were observed in the curcumin group, whereas 17.5% of individuals in the placebo group experienced an increase in liver fat content [39]. The findings by Cicero et al. demonstrated similar outcomes in relation to the parameters of fatty liver index and liver steatosis [40]. Jazayeri-Tehrani et al. indicated that the administration of nano-curcumin to overweight/obese patients with NAFLD resulted in improved liver function and a reduction in fatty liver severity [41]. Nouri-Vaskeh et al. discovered that curcumin supplementation had positive impacts on disease activity scores and the severity of cirrhosis [37]. Panahi et al. [42] elucidated that the administration of curcuminoids together with piperine resulted in a considerable reduction in the severity of NAFLD, a phenomenon that was found to be statistically distinct in comparison to the placebo group. Moreover, it was observed that individuals in the placebo group did not exhibit any alterations from their initial baseline measurements [42]. The ultrasonographic findings of Panahi et al. [43] showed a significant improvement of NAFLD severity in 75.0% of subjects in the curcumin group, compared to only 4.7% in the control group. Additionally, the occurrence of increased liver fat content was lower in the curcumin group at 4.5%, while 25.6% of subjects in the placebo group experienced an increase. Analysis between the groups demonstrated notable enhancements in hepatic vein flow, portal vein diameter, and liver volume in the curcumin group compared to the placebo group [44].

### 2.6. Green Tea

Green tea is defined as the unfermented leaves of *Camellia sinensis* (L.) Kuntze. It is the second most popular drink, after water, especially in Southeast Asia. Consumption of tea is related to flavanols and other polyphenols known as catechins, but also caffeine, which may be health beneficial [45]. Epigallocatechin-3-gallate (EGCG) is the major present catechin in green tea [46]. The medical properties are as follows: cytotoxic, anti-diarrheal, anti-microbial, antioxidative and anti-inflammatory, anti-obesity, and anti-diabetic [47].

A total of 253 patients diagnosed with NAFLD and NASH were administered green tea catechin at dosages ranging from 550 to 1080 mg per day, or a green tea extract of 500 mg standardized for its epigallocatechin gallate (EGCG) content (31.43% to 52.6%), for a duration of 12 to 24 weeks (Table S5). These patients were from two different locations in Japan, as well as from Iran and Pakistan [48]. The impact of the treatment was assessed based on various liver parameters, including ALT levels, as measured in all five studies, as well as AST in four studies, GGT in one study, and ALP in one study. Results across all clinical trials consistently showed a significant decrease in these liver parameters. It is important to note that a study conducted by Pezeshki et al. (2016) revealed a reduction in hepatic outcomes among placebo groups, suggesting a potential correlation with lifestyle modifications [45].

Fukuzawa et al. shown that green tea catechins, in combination with structured dietary plans and exercise regimens, can improve key anthropometric parameters in ultrasonographic findings, such as the ratio of visceral fat to subcutaneous fat (V/S) area, as well as the liver-to-spleen (L/S) ratio. This suggests that these interventions may help to prevent the progression of NASH by utilizing their antioxidant and anti-inflammatory properties to reduce oxidative stress in patients with NASH [46]. Hussain et al. [48] revealed a significant 67.5% reduction in fatty liver changes in ultrasounds among participants who consumed green tea extract, in comparison to those who received a placebo showing only a 25% regression. This suggests that green tea extract may play a promising role in inhibiting the advancement of NAFLD [48]. Sakata et al. [49] discovered that individuals who consumed a high-density catechin supplement experienced a significant decrease in body fat compared to those taking a placebo or low-density catechin supplement over a period of 12 weeks. Furthermore, all participants in the high-density catechin group exhibited a notable improvement in their liver-to-spleen CT attenuation ratio as compared to those in the placebo and low-density catechin groups after the same duration of consumption [49].

### 2.7. Schisandra

Schisandra (*Schisandra chinensis* (Turcz.) Baill.) fruits are traditional East Asian medicine used in obesity and diabetes [50]. The fruits are rich in lignans called schisandrins with antioxidative, anti-inflammatory, and cytotoxic properties [51]. Schisandrin B reduced hepatic lipids in rat studies [52]. The probable mechanism of action of schisandra fruits involves the inhibition of adipogenesis in preadipocyte cells, as noted in the in vivo studies [50].

A total of 100 patients (Table S6) with mild liver injury or dysfunction were included in two studies to assess the impact of schisandra on hepatic outcomes. The trials were conducted in Indonesia and Taiwan. Patients were administered a schisandra extract (7.5 mg daily) or schisandrin B (0.24–0.48 mg) combined with sesamin.

The first study, conducted by Ardiyanto et al., found that the schisandra extract was just as effective as a traditional herbal medicine (used in the placebo group) in treating liver disturbances, as evidenced by confirmed results in ALT and AST levels [51].

The second study showed a significant decrease in liver parameters among patients in the intervention group who were given a combination of schisandrin B and sesamin. Moreover, a marked improvement in the ultrasonographic examinations of fatty liver and liver function was observed. However, it should be noted that this study did not establish the effectiveness of schisandrin B on its own, but rather in combination with sesamin [52].

### 2.8. Silymarin

Silymarin is a flavonolignan fraction of milk thistle (*Silybum marianum* (L.) Gaertner), comprising mainly silybin, silydianin, silychristin, and isosilybin. The seeds of milk thistle have a long history of use in liver disorders [53]. The use of milk thistle (silymarin) is believed to have several potential benefits in the field of hepatology. These include preventing the entry of toxins such as alcohol, carbon tetrachloride, and heavy metals into liver cells, stimulating protein synthesis for liver regeneration, acting as an antioxidant to scavenge free radicals, and modulating the immune response. Research studies have shown hepatoprotective effects of silymarin in cases of fatal fulminant hepatic failure caused by *Amanita phalloides* mushroom poisoning in both animal and human subjects, even when administered after exposure. While some randomized controlled trials have reported inconclusive results, particularly in cases of alcoholic liver disease, chronic hepatitis B (HBV), or hepatitis C (HCV) infections, further research is needed to fully understand the potential benefits of milk thistle/silymarin supplementation in these conditions [54].

A total of 676 patients diagnosed with various liver conditions, including NAFLD, NASH, acute/chronic hepatitis, cirrhosis, and other liver disorders, were included in the studies (Table S7) to evaluate the impact of silymarin on liver health outcomes [53,54,55,56,57,58,59,60]. The patients ranged in age from 29 to 63 years old. The studies were conducted in multiple countries, including Iran, Australia, Spain, Egypt, Finland, and Denmark. Patients were administered varying dosages of milk thistle extract—with some standardized for silymarin amounts ranging from 140 to 600 mg daily, with a standard dose of 420 mg for a follow-up period of 4 weeks to 12 months. While three out of the eight studies did not demonstrate significant improvements in liver outcomes, one study conducted by Fried et al. (2012) revealed that a higher dose of silymarin, specifically 720 mg daily, was more effective in reducing liver parameters compared to the standard 420 mg dosage [55]. The histological examination conducted by Salmi and Sarna (1982) revealed a statistically significant improvement in the silymarin group compared to the placebo group in terms of fatty liver transformation and the score of hepatitis [56].

## 3. Algae and Probiotics

### 3.1. Chlorella

Chlorella (*Chlorella vulgaris* Beijer.) is a unicellular green algae known worldwide as a functional food and used as a traditional medicine in Asia [61]. It is rich in choline, β-carotene, and amino acids. Numerous essential vitamins and minerals are present in chlorella, including thiamine, riboflavin, niacin, pantothenic acid, pyridoxine, biotin, folic acid, cobalamin, ascorbic acid, tocopherols, sodium, potassium, calcium, magnesium, phosphorous, copper, zinc, manganese, iodine, and iron. Its bioactive compounds, mainly pigments, include chlorophyll, beta-carotene, astaxanthin, canthaxanthin, violaxanthin, lutein, and pheophytin, essential for many bioactivities [17]. In animals, many health-beneficial effects were found, such as anti-microbial, cytotoxic, and anti-diabetic effects, but also improvements in liver function were noted. However, there has been a limited number of trials on human subjects [62]. The mechanism underlying the improvement of hepatic outcomes might be related to the weight loss effects of chlorella, observed as a decrease in hepatic triglyceride content and gluconeogenesis, which eventually results in a reduction of liver parameters [17].

In a total of 271 patients diagnosed with NAFLD or hepatitis C, the interventions involved the administration of chlorella in powdered tablets, ranging from 1 to 3 g daily for a period of 8 to 12 weeks. The majority of the studies (Table S8), four out of five, were conducted in Iran, with one study originating from the USA [63]. All five studies demonstrated overall enhancements in liver parameters, with the exception of Panahi et al. (2012) where a placebo group administered with metformin showed a greater decrease in ALT and ALP serum levels compared to the chlorella intervention group [17]. It is important to note that only AST levels showed improvement in this particular study. Additionally, Ebrahimi et al. (2014) conducted a notable study which exhibited some reduction in liver parameters in the placebo group (which received only vitamin E supplementation) [61]. A study by Azocar and Diaz (2013) showed a statistical decrease in HCV viral load. The HCV RNA levels were confirmed in 69.23% of the participants [63]. Other studies have not assessed physical liver function [17,61,62,64,65].

### 3.2. Spirulina

Spirulina (*Arthrospira platensis*) is a green–blue, multicellular microscopic algae mostly found in alkaline lakes. This filamentous cyanobacterium is cultivated in human-controlled conditions for global consumption [66]. It has been used for thousands of years but recently is becoming more popular, as it is a rich source of essential nutrients including vitamins such as thiamine, riboflavin, niacin, cobalamin, ascorbic acid, cholecalciferol, and tocopherol; minerals including potassium, calcium, iron, magnesium, phosphorus, selenium, sodium, and zinc; pigments (e.g., chlorophyll A, xanthophylls, β-carotene, zeaxanthin, canthaxanthin, diatoxanthin, β-cryptoxanthin, C-phycocyanin, and allophycocyanin); and enzymes (such as lipase), as well as eight crucial amino acids. The ratio of saturated palmitic acid in the total fatty acids is 25.8%, while the percentage of γ-linolenic acid in the total fatty acids is 40.1%. Spirulina serves as a valuable dietary supplement to address essential fatty acid deficiencies and provide antioxidants (including chlorophyll, phycocyanin pigments, and phenolic compounds which equate to 26.64 mg of gallic acid per gram of extract) [67]. Spirulina has revealed positive outcomes in trials of NAFLD, diabetes, arterial hypertension, and hypercholesterolemia [65].

A total of 128 patients with NAFLD or chronic viral hepatitis were included in a study (Table S9) analyzing the effect of spirulina on liver function. Two studies were conducted in Iran, with additional studies from Romania, Mexico, and Greece. Spirulina doses ranged from 1 to 4.5 g in the form of powdered tablets, taken daily for 4 weeks to 6 months. Only two out of the five studies included a placebo group. Overall, significant improvements in hepatic liver enzymes were reported in all studies, with the exception of the study by Baicus and Tănăsescu (2002) which focused on patients with viral chronic hepatitis (with etiology of infection with hepatitis virus B or C), potentially indicating different results compared to those with NAFLD [68]. Notably, a case report from Mexico involving three patients with NAFLD demonstrated significant impacts on liver parameters, observed as a notable enhancement in liver ultrasonography. All patients exhibited an improvement in liver ultrasonography related to NAFLD. Two patients showed a decrease in parenchyma heterogeneity and the third patient experienced a complete resolution of the “bright liver” phenomenon. However, the results can only be treated as preliminary. The authors opted not to perform liver biopsies due to the high risk involved for ambulatory patients and the limited technical and medical resources available in the laboratory to manage potential complications [69]. On the contrary, the study by Mazokopakis et al. (2014) did not reveal any significant alterations in sonographic results within the two groups under observation [67].

### 3.3. Probiotics

Probiotics are traditionally described as a variety of bacteria in the intestine with a full spectrum of health benefits for the host. The mechanism of how those microbes work in patients with NAFLD is not fully understood [70]. It is suggested that it might be connected to the interaction of eradicating pathogenic bacteria in the intestine [71]. Potential mechanisms of action related to liver disease involve the reduction of circulating levels of pro-inflammatory mediators, such as TNF-α, IL-1β, or IL-6. Additionally, there may be a decrease in hepatocyte ballooning and the development of liver lobular fibrosis [72]. Intestinal dysbiosis is called intestinal bacterial overgrowth (SIBO). This acts via the blocking of adhesion sites, degradation of toxin receptors, and absorption of gut-derived lipopolysaccharides [73].

A total of 682 patients diagnosed with NAFLD, NASH, minimal hepatic encephalopathy, or obesity-related liver disease in children were included in the reviewed studies, evaluating the efficacy of probiotics and prebiotics on liver parameters. The studies (Table S10) originated from multiple countries, with three from Iran, two from India and Italy, and single ones from Spain, Egypt, Ukraine, the USA, Brazil, and Hong Kong [64,70,71,72,73,74,75,76,77,78,79,80,81]. The interventions in the studies varied, with three utilizing a single strain of bacteria, including *Lactobacillus rhamnosus* (12 billion CFU) (dsm2 21690), *L. acidophilus* (2 billion CFU) (DSM B3208, DSM 24735), or *Bifidobacterium longum* (2 billion CFU) (DSM 24736), as well as combinations such as *L. bulgaricus* and *Streptococcus thermophilus* (500 million CFU) (DSM 24731) or various multi-strain formulations ranging from 15 million to 112.5 billion CFU.

The notable research conducted by Javedi et al. (2017) indicated a more pronounced decrease in liver parameters within the probiotic plus prebiotic group when compared to the probiotic group alone [72]. In addition, a study by Malaguarnera et al. (2012) revealed a considerable decrease in liver parameters within the placebo group, which the authors attributed to lifestyle interventions within both groups [75].

Subsequent liver biopsies were conducted by Duseja et al. (2019) one year post-initiation in a cohort of 10 patients (52.6%) within the probiotic group and five patients (25%) within the placebo group. Significant enhancements in hepatocyte ballooning, lobular inflammation, and NAS score were observed at the one-year mark in the probiotic group compared to baseline levels. Furthermore, significant improvements were noted in the NAS score, hepatocyte ballooning, and hepatic fibrosis in the probiotic group when compared to the placebo group [76]. Famouri et al. (2017) discovered that after their trial, normal liver sonography was reported in 17 (53.1%) of the patients in the intervention group and five (16.5%) of the patients in the placebo group. This demonstrates the beneficial effect of probiotic supplementation [77]. The research conducted by Javadi et al. (2017) demonstrated that the use of a probiotic (capsules containing *B. longum* and *L. acidophilus* at a dosage of 2 × 10^7^ CFU per day), a prebiotic (group 2, high-performance inulin administered at a dosage of 10 g per day), and a combination of the probiotic and prebiotic (group 3) led to improvements in the severity of fatty liver in patients with NAFLD [72]. Malaguarnera et al. (2012) demonstrated that the combination of *B. longum* supplementation with prebiotics and lifestyle modifications significantly reduced steatosis and the NASH activity index compared to lifestyle modification alone [75]. After the intervention, Wong et al. (2013) found that liver fat decreased by a mean of 7.7%, as observed through proton-magnetic resonance spectroscopy. In contrast, the usual-care group showed a static hepatic fatty content with a mean reduction of only 1.0% [78]. On the contrary, Monem (2017) demonstrated that probiotic treatment did not lead to any notable changes in abdominal ultrasound results [79].

## 4. Other Natural Products

### 4.1. Essential Phospholipids

Phospholipids are the main components of mammalian cells and lipoproteins [82]. They are necessary for the formation, regeneration, and movement of membrane structures and the restoration of membranes’ structure and fluidity, the inhibition of fibrotic processes, and the modification of lipid metabolism, and therefore have anti-inflammatory and antioxidant properties [83]. Essential phospholipids (EPLs) are the purified fraction obtained from soybeans and are widely used in liver diseases [84].

In total, 705 patients with NAFLD and NASH were enrolled in trials involving essential phospholipids for the improvement of liver enzymes (Table S11) [82,83,84,85,86,87,88,89]. Among these trials, only three out of eight were randomized controlled trials with a placebo group, while the rest were open-label studies. The participants in these studies ranged in age from 41 to 59, with a BMI between 28.2 and 29.3. The studies were conducted in various countries including China (three studies), Poland, India, Russia, the United Arab Emirates, and Sweden.

All studies utilized the same dosage of 1800 mg of essential phospholipids per day, administered in divided doses. The duration of follow-up ranged from 4 to 48 weeks. Results from all studies consistently showed a decrease in liver parameters. One study by Yin and Kong (2000) included a comparative analysis with metformin [85]. However, the effectiveness of different dosages could not be assessed as all studies used the same dosing regimen.

The ultrasound studies conducted by Poongothai et al. (2005) demonstrated that hepatic echotexture improved in 54.5% of the study subjects following EPL treatment. Conversely, no change was observed in 40.9% of the participants, and only one patient (4.5%) experienced a worsening of hepatic echotexture [86]. In a study conducted by Gonciarz et al. (1988), it was demonstrated that EPL exhibited significant effectiveness in reducing liver size, likely due to its ability to decrease liver fat content. This finding was corroborated by histological evaluations of post-treatment biopsies, which indicated the absence of fatty infiltration in four patients from the Essentiale Forte group exclusively [82].

### 4.2. Vitamin D

Cholecalciferol (vitamin D) is crucial for maintaining phosphate and calcium balance [90]. Those subjects affected by D-deficiency are more likely to develop impaired glucose tolerance and increase the amount of free fat in their liver which is directly linked to the development of NAFLD [90,91]. In recent decades, research has conclusively demonstrated the role of vitamin D in modulating various immune–inflammatory and metabolic processes [92]. Subsequently, there has been a focus on studying the interaction between the active form of vitamin D—1,25-dihydroxy-vitamin D—and the vitamin D receptor (VDR) in relation to disruptions in metabolic pathways within various organs and tissues. This includes but is not limited to the skeletal muscle, adipose tissue, and liver, which are crucial players in metabolic regulation [93].

A total of 461 individuals with NAFLD were included in the studies to assess the effectiveness of vitamin D on liver parameters (Table S12) [90,91,92,93,94]. The dosage of vitamin intake ranged from 800 IU daily to 50,000 IU weekly when taken orally, or from 200,000 IU to 600,000 IU in a single intramuscular injection when administered parenterally. The average age of the participants ranged from 38.3 to 54, with a BMI range from 27.0 to 31.2. The studies were conducted in Iran, Egypt, and India, with follow-ups ranging from 10 weeks to 12 months. Four out of five studies demonstrated statistical improvements in liver function, with no observed correlation between dosage and effectiveness.

Notably, a study by Shidfar et al. (2019) highlighted the synergistic effect of vitamin D combined with calcium on liver enzymes ALT and AST compared to vitamin D alone. This suggests that a combination of both substances may be beneficial in maintaining optimal levels of vitamin D and calcium in the body [92].

Sakpal et al. (2017) discovered that there was no variance in the baseline level of steatosis and hepatic fibrosis stage between the two groups who received either vitamin D or a placebo [93]. Gad et al. found that a single monthly intramuscular dose of 200,000 IU cholecalciferol effectively improved laboratory and fibroscan parameters of the liver in patients with NAFLD [91].

## 5. Discussion

Our review comprised thirteen natural products often used in supporting liver disorders as herbal drugs or dietary supplements. The presented results indicate that some of the presented natural products might satisfactorily improve hepatic outcomes in NAFLD, NASH, and other liver disorders. None of the studies indicated a significant toxic effect of these non-synthetic remedies on liver function.

**Figure 1:**
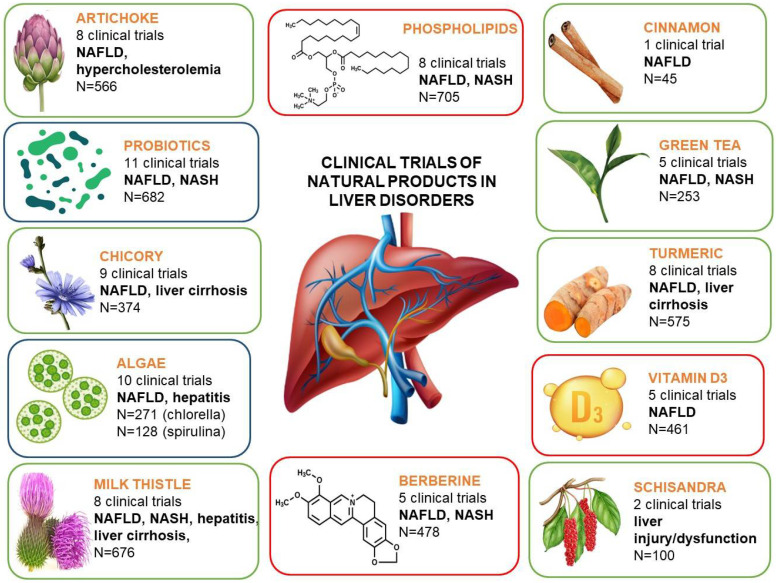
Summary of the clinical trials of natural products presented in this review.

Apart from the well-known natural products, like silymarin, artichoke, or essential phospholipids, often used by patients with liver disorders, some new, often surprising candidates for liver support can be indicated, based on the results of the studies cited in this review (Figure 1). These may particularly include the representatives of algae, namely chlorella and spirulina, but also the probiotics. The latter candidate seems to be of the highest interest, as the clinical studies on probiotic use in liver disorders comprised almost 600 patients/participants (as compared to the often-used silymarin, with 676 participants), and were properly designed, which could be a solid evidence-based argument for their use. The studies concerning algae, despite the smaller number of participants, revealed interesting potential of both chlorella and spirulina in supporting liver disorders, and these studies deserve continuation to deliver more clear evidence.

The studies presented in this review have some limitations. Firstly, using hepatic parameters such as ALT, AST, and ALP as direct markers for NAFLD diagnosis is not obvious, and there are no other effective measure outcomes to use in clinical trials. The number of studies performed and, in particular, the sizes of the groups included within the studies were relatively low or not satisfactory. Additionally, some studies lacked a control/placebo group, while in a few the reference group was receiving a drug affecting the metabolism (e.g., metformin), which might have disturbed the final results and conclusions drawn. Moreover, the majority of the studies were carried out in Iran; therefore, the effect of polymorphism was not investigated. The effect of sex was not investigated in this review because most trials have not quantified participants by male or female. Concerning the forms and doses used in the clinical trials cited, the direct comparison of the results for particular natural products was difficult, due to the high diversity of the examined products, e.g., an extract, standardized extract, pulverized natural product, or an isolated fraction containing the dominant compounds. This was mainly observed for artichoke, chicory, and silymarin, where the unification of the products used in the studies needs improvement in the future. In contrast, the studies concerning essential phospholipids were easier to compare, as the dose used was the only difference between the studies. On the other hand, among the studies performed on essential phospholipids, the vast majority (six out of eight) were open label studies without a control group, which impairs their credibility.

## 6. Conclusions

In conclusion, the results of the studies included in this review indicate the improvement in some liver outcomes’ measurements after the use of artichoke, berberine, chlorella, chicory, green tea, probiotics, phospholipids, schisandra, silymarin, spirulina, and vitamin D in liver injury and NAFLD. In contrast, other studies with cinnamon and turmeric have not significantly changed liver enzymes in response to herbal supplementation. However, it is important to underline that these studies refer to the effects of the mentioned products in patients with already identified liver diseases, while their hepatoprotective or perhaps hepatoprophylactic potential has not been assessed during the actual exposure to hepatotoxic agents. These results thus need confirmation in future larger-scale meta-analyses to seek the optimally effective doses of the examined natural products on liver enzymes, with longer supplementation periods, with NAFLD of varying severities, and with the assessment of other liver parameters (liver size, hepatic steatosis, and hepatic fibrosis).

## Data Availability

No new data were created or analyzed in this study. Data sharing is not applicable to this article.

## Supplementary Materials

The following supporting information can be downloaded at https://www.mdpi.com/article/10.3390/plants13141985/s1; Figure S1: Flowchart of the search strategy; Table S1: Clinical trials of hepatoprotective effect of artichoke; Table S2: Clinical trials of hepatoprotective effect of berberine; Table S3: Clinical trials of hepatoprotective effect of chicory; Table S4: Clinical trials of hepatoprotective effect of turmeric/curcumin; Table S5: Clinical trials of hepatoprotective effect of green tea; Table S6: Clinical trials of hepatoprotective effect of schisandra; Table S7: Clinical trials of hepatoprotective effect of milk thistle/silymarin; Table S8: Clinical trials of hepatoprotective effect of chlorella; Table S9: Clinical trials of hepatoprotective effect of spirulina; Table S10: Clinical trials of hepatoprotective effects of probiotics; Table S11: Clinical trials of hepatoprotective effect of phospholipids; Table S12: Clinical trials of hepatoprotective effect of vitamin D.

## References

[1] Ward F.M., Daly M.J., Walker R., Edwards C. (1999). Hepatic Disease. Clinical Pharmacy and Therapeutics.

[2] Hadzagic-Catibusic F., Hasanbegovic E., Melunovic M., Zubcevic S., Uzicanin S. (2017). Effects of carbamazepine and valproate on serum aspartate aminotransferase, alanine aminotransferase and gamma-glutamyltransferase in children. Med. Arch..

[3] Devarbhavi H., Asrani S.K., Arab J.P., Nartey Y.A., Pose E., Kamath P.S. (2023). Global burden of liver disease: 2023 update. J. Hepatol..

[4] Adewusi E., Afolayan A. (2010). A review of natural products with hepatoprotective activity. J. Med. Plants Res..

[5] Bedogni G., Bellentani S., Miglioli L., Masutti F., Passalacqua M., Castiglione A., Tiribelli C. (2006). The Fatty Liver Index: A simple and accurate predictor of hepatic steatosis in the general population. BMC Gastroenterol..

[6] Ballestri S., Zona S., Targher G., Romagnoli D., Baldelli E., Nascimbeni F., Roverato A., Guaraldi G., Lonardo A. (2016). Nonalcoholic fatty liver disease is associated with an almost twofold increased risk of incident type 2 diabetes and metabolic syndrome. Evidence from a systematic review and meta-analysis. J. Gastroenterol. Hepatol..

[7] Ringehan M., McKeating J.A., Protzer U. (2017). Viral hepatitis and liver cancer. Phil. Trans. R. Soc. B.

[8] GBD Collaborators (2017). Disease and Injury Incidence and Prevalence Collaborators Global, regional, and national incidence, prevalence, and years lived with disability for 354 diseases and injuries for 195 countries and territories, a systematic analysis for the Global Burden of Disease Study. Lancet.

[9] Yang K., Chen J., Zhang T., Yuan X., Ge A., Wang S., Xu H., Zeng L., Ge J. (2022). Efficacy and safety of dietary polyphenol supplementation in the treatment of non-alcoholic fatty liver disease: A systematic review and meta-analysis. Front. Immunol..

[10] Andersen T., Gluud C., Franzmann M.-B., Christoffersen P. (1991). Hepatic effects of dietary weight loss in morbidly obese subjects. J. Hepatol..

[11] Targher G., Tilg H., Byrne C.D. (2021). Non-alcoholic fatty liver disease: A multisystem disease requiring a multidisciplinary and holistic approach. Lancet Gastroenterol. Hepatol..

[12] Deshwal N., Sharma A., Sharma P. (2011). Review on hepatoprotective plants. Int. J. Pharm. Sci. Rev. Res..

[13] Panahi Y., Kianpour P., Mohtashami R., Atkin S.L., Butler A.E., Jafari R., Badeli R., Sahebkar A. (2018). Efficacy of artichoke leaf extract in non-alcoholic fatty liver disease: A pilot double-blind randomized controlled trial. Phytotherapy Res..

[14] Barrat E., Yassine Z., Pascal S., Chauveau P., Maudet C. (2013). Effect on LDL-cholesterol of a large dose of a dietary supplement with plant extracts in subjects with untreated moderate hypercholesterolaemia: A randomised, double-blind, placebo-controlled study. Eur. J. Nutr..

[15] Englisch W., Beckers C., Unkauf M., Ruepp M., Zinserling V. (2020). Efficacy of Artichoke dry extract in patients with hyperlip-oproteinemia. Arzneimittelforschung.

[16] Majnooni M.B., Ataee M., Bahrami G., Heydarpour F., Aneva I.Y., Farzaei M.H., Ahmadi-Juoibari T. (2021). The effects of co-administration of artichoke leaf extract supplementation with metformin and vitamin E in patients with nonalcoholic fatty liver disease: A randomized clinical trial. Phytother. Res..

[17] Panahi Y., Ghamarchehreh M.E., Beiraghdar F., Zare R., Jalalian H.R., Sahebkar A. (2012). Investigation of the effects of Chlorella vulgaris supplementation in patients with non-alcoholic fatty liver disease: A randomized clinical trial. Hepato-Gastroenterology.

[18] Musolino V., Gliozzi M., Bombardelli E., Nucera S., Carresi C., Maiuolo J., Mollace R., Paone S., Bosco F., Scarano F. (2020). The synergistic effect of Citrus bergamia and Cynara carduncu-lus extracts on vascular inflammation and oxidative stress in non-alcoholic fatty liver disease. J. Tradit. Compl. Med..

[19] Cicero A., Fogacci F., Bove M., Giovannini M., Veronesi M., Borghi C. (2019). Short-Term Effects of Dry Extracts of Artichoke and Berberis in Hypercholesterolemic Patients without Cardiovascular Disease. Am. J. Cardiol..

[20] Fogacci F., Rizzoli E., Giovannini M., Bove M., D’addato S., Borghi C., Cicero A.F.G. (2022). Effect of Dietary Supplementation with Eufortyn^®^ Colesterolo Plus on Serum Lipids, Endothelial Reactivity, Indexes of Non-Alcoholic Fatty Liver Disease and Systemic Inflammation in Healthy Subjects with Polygenic Hypercholesterolemia: The ANEMONE Study. Nutrients.

[21] Rangboo V., Noroozi M., Zavoshy R., Rezadoost S.A., Mohammadpour A. (2016). The Effect of Artichoke Leaf Extract on Ala-nine Aminotransferase and Aspartate Aminotransferase in the Patients with Nonalcoholic Steatohepatitis. Int. J. Hepatol..

[22] Nejati L., Movahedi A., Salari G.R., Moeineddin R., Nejati P. (2022). The Effect of Berberine on Lipid Profile, Liver Enzymes, and Fasting Blood Glucose in Patients with Non-alcoholic Fatty Liver Disease (NAFLD): A Randomized Controlled Trial. Med. J. Islam. Repub. Iran.

[23] Yan H.-M., Xia M.-F., Wang Y., Chang X.-X., Yao X.-Z., Rao S.-X., Zeng M.-S., Tu Y.-F., Feng R., Jia W.-P. (2015). Efficacy of Berberine in Patients with Non-Alcoholic Fatty Liver Disease. PLoS ONE.

[24] Cao Y., Cai W., Wei L., Zhang L., Fang Y. (2016). Clinical observation on the Berberine plus metformin in treatment of type 2 diabetes complicated by nonalcoholic fatty liver disease. Modern. Prev. Med..

[25] Harrison S., Gunn N., Neff G., Kohli A., Liu L., Flyer A., Goldkind L., Bisceglie A. (2021). A phase 2, proof of concept, randomised controlled trial of berberine ursodeoxycholate in patients with presumed non-alcoholic steatohepatitis and type 2 diabetes. Nat. Commun..

[26] Bai B., Zheng B., Zhang R.D., Wei J. (2011). Effects of berberine on insulin resistance and serum adiponectin of nonalcoholic fatty liver patients. Pract. Geriatr..

[27] Marzban M., Bahrami M., Kamalinejad M., Tahamtan M., Khavasi N., Haji-Monfared M., Jameshorani M. (2022). The therapeutic effects of chicory seed aqueous extract on cardio-metabolic profile and liver enzymes in nonalcoholic fatty liver disease; a double blind randomized clinical trial. Immunopathol. Persa.

[28] Parveen F., Siddique M.A., Quamri M.A., Khaleel A., Nayak T., Mariyam A. (2020). Cichorium Intybus and Solanum Nigrum leave juice (Murawwaquain) reduces raised Liver Enzymes and improved conditions associated with Hepatobiliary Diseases: A Single Blinded, Pre and Post Analytical Study. Int. J. Res. Anal. Rev..

[29] Hassani A., Ansari R., Mazani A. (2016). Effect of 8 weeks of aerobic training and using chicory extractive supplementation on serum levels of ALT and AST enzymes in women with fatty liver. Iran. J. Obstetr. Gynecol. Infertil..

[30] Nikkhajoei M., Choopani R., Tansaz M., Hashem-Dabaghian F. (2016). Efficacy of Cichorium intybus on Alanine Aminotransferase in Nonalcoholic Fatty Liver Disease: A Randomised Double-blind Clinical Trial with Placebo. Biol. Forum Int. J..

[31] Ghaffari A., Rafraf M., Navekar R., Sepehri B., Asghari-Jafarabadi M., Ghavami S.-M. (2019). Turmeric and chicory seed have beneficial effects on obesity markers and lipid profile in non-alcoholic fatty liver disease (NAFLD). Int. J. Vitam. Nutr. Res..

[32] Huseini H., Alavian S., Heshmat R., Heydari M.R., Abolmaali K. (2005). The efficacy of Liv-52 on liver cirrhotic patients: A randomized, double-blind, placebo-controlled first approach. Phytomedicine.

[33] Asl Z.S., Malekirad A., Abdollahi M., Bakhshipour A., Dastjerdi H. (2014). Mostafalou of the Mixture of Cichorium intybus L and Cinnamomum zeylanicum on Hepatic Enzymes Activity and Biochemical Parameters in Patients with Nonalcoholic Fatty Liver Disease. Health.

[34] Elmieh A., Rafizadeh B., Khanbabakhani H. (2020). Effect of aerobic interval training and consumption of chicory extract on levels of liver enzymes in obese boys with non-alcoholic fatty liver. J. Appl. Exerc. Physiol..

[35] Faraji S., Azar M.R.M.H., Alizadeh M. (2022). Brewed chicory leaf consumption has unexpected side effects along beneficial effects on liver enzymes in non-alcoholic fatty liver disease patients. J. Herb. Med..

[36] Askari F., Rashidkhani B., Hekmatdoost A. (2014). Cinnamon may have therapeutic benefits on lipid profile, liver enzymes, insulin resistance, and high-sensitivity C-reactive protein in nonalcoholic fatty liver disease patients. Nutr. Res..

[37] Nouri-Vaskeh M., Mahdavi M.A., Afshan H., Alizadeh L., Zarei M. (2020). Effect of curcumin supplementation on disease severity in patients with liver cirrhosis: A randomized controlled trial. Phytother. Res..

[38] Saadati S., Hatami B., Yari Z., Shahrbaf M.A., Eghtesad S., Mansour A., Poustchi H., Hedayati M., Aghajanpoor-Pasha M., Sadeghi A. (2019). The effects of curcumin supplementation on liver enzymes, lipid profile, glucose homeostasis, and hepatic steatosis and fibrosis in patients with non-alcoholic fatty liver disease. Eur. J. Clin. Nutr..

[39] Rahmani S., Asgary S., Askari G., Keshvari M., Hatamipour M., Feizi A., Sahebkar A. (2016). Treatment of Non alcoholic Fatty Liver Disease with Curcumin: A Randomized Placebo controlled Trial. Phytother. Res..

[40] Cicero A.F.G., Sahebkar A., Fogacci F., Bove M., Giovannini M., Borghi C. (2020). Effects of phytosomal curcumin on anthropometric parameters, insulin resistance, cortisolemia and non-alcoholic fatty liver disease indices: A double-blind, placebo-controlled clinical trial. Eur. J. Nutr..

[41] Jazayeri-Tehrani S.A., Mahdi Rezayat S., Mansouri S., Qorbani M., Moayed-Alavian S., Daneshi-Maskooni M., Hosseinzadeh-Attar M.-J. (2019). Nano-curcumin improves glucose indices, lipids, inflammation, and Nesfatin in overweight and obese patients with non-alcoholic fatty liver disease (NAFLD): A double-blind randomized placebo controlled clinical trial. Nutr. Metab..

[42] Panahi Y., Kianpour P., Mohtashami R., Jafari R., Simental-Mendía L.E., Sahebkar A. (2017). Efficacy and Safety of Phytosomal Curcumin in Non-Alcoholic Fatty Liver Disease: A Randomized Controlled Trial. Drug Res..

[43] Panahi Y., Hosseini M.S., Khalili N., Naimi E., Simental-Mendía L.E., Majeed M., Sahebkar A. (2016). Effects of curcumin on serum cytokine concentrations in subjects with metabolic syndrome: A post-hoc analysis of a randomized controlled trial. Biomed. Pharmacother..

[44] Jarhahzadeh M., Alavinejad P., Farsi F., Husain D., Rezazadeh A. (2021). The effect of turmeric on lipid profile, malondialdehyde, liver echogenicity and enzymes among patients with nonalcoholic fatty liver disease: A randomized double blind clinical trial. Diabetol. Metab. Syndr..

[45] Askari G., Pezeshki A., Safi S., Feizi A., Karami F. (2016). The effect of green tea extract supplementation on liver enzymes in patients with nonalcoholic fatty liver disease. Int. J. Prev. Med..

[46] Fukuzawa Y., Kapoor M., Yamasaki P., Okubo K., Hotta T., Juneja L. (2014). Effects of green tea catechins on non-alcoholic steatohepatitis (NASH) patients. J. Funct. Foods.

[47] Tabatabaee S.M., Alavian S.M., Ghalichi L., Miryounesi S.M., Mousavizadeh K., Jazayeri S., Vafa M.R. (2017). Green tea in non-alcoholic fatty liver disease: A double blind randomized clinical trial. Hepat. Mon..

[48] Mazhar Hussain M.H., Habib-ur-Rehman H.U.R., Lubna Akhtar L.A. (2017). Therapeutic benefits of green tea extract on various parameters in non-alcoholic fatty liver disease patients. Pak. J. Med. Sci..

[49] Sakata R., Nakamura T., Torimura T., Ueno T., Sata M. (2013). Green tea with high-density catechins improves liver function and fat infiltration in non-alcoholic fatty liver disease (NAFLD) patients: A double blind placebo controlled study. Int. J. Mol. Med..

[50] Li X., Liang J., Zhang D.-Y., Kuang H.-X., Xia Y.-G. (2021). Low-polymerization compositional fingerprinting for characterization of Schisandra polysaccharides by hydrophilic interaction liquid chromatography-electrospray mass spectrometry. Int. J. Biol. Macromol..

[51] Ardiyanto D., Zulkarnain Z., Astana P., Triyono A., Novianto F., Fitriani U., Nisa U., Mana T. (2021). Efficacy of hepatoprotector jamu formula (combination of Curcuma longa, Curcuma xanthorrhiza, and Taraxacumofficinale) compared to Fructus schizandrae extract in mild liver injury: A randomized controlled trial. IOP Conf. Ser. Earth Environ. Sci..

[52] Chiu H.-F., Chen T.-Y., Tzeng Y.-T., Wang C.-K. (2012). Improvement of Liver Function in Humans Using a Mixture of *Schisandra* Fruit Extract and Sesamin. Phytotherapy Res..

[53] Hashemi S.J., Hajiani E., Sardabi E.H. (2009). A placebo-controlled Trial of Silymarin in Patients with Nonalcoholic Fatty Liver Disease. Hepatitis.

[54] Gordon A., Hobbs D.A., Bowden D.S., Bailey M.J., Mitchell J., Francis A.J., Roberts S.K. (2006). Effects of Silybum marianum on serum hepatitis C virus RNA, alanine aminotransferase levels and well-being in patients with chronic hepatitis. J. Gastroenterol. Hepatol..

[55] Fried M.W., Navarro V.J., Afdhal N., Belle S.H., Wahed A.S., Hawke R.L., Doo E., Meyers C.M., Reddy K.R. (2012). Silymarin in NASH and C Hepatitis (SyNCH) Study Group. Effect of silymarin (milk thistle) on liver disease in patients with chronic hepatitis C unsuccessfully treated with interferon therapy: A randomized controlled trial. JAMA.

[56] Salmi H.A., Sarna S. (1982). Effect of Silymarin on Chemical, Functional, and Morphological Alterations of the Liver, A Double-Blind Controlled Study. Scand. J. Gastroenterol..

[57] Aller R., Izaola O., Gómez S., Tafur C., González G., Berroa E., Mora N., González J.M., De Luis D.A. (2015). Effect of silymarin plus vitamin E in patients with non-alcoholic fatty liver disease. A randomized clinical pilot study. Eur. Rev. Med. Pharmacol. Sci..

[58] El-Kamary S.S., Shardell M.D., Abdel-Hamid M., Ismail S., El-Ateek M., Metwally M., Mikhail N., Hashem M., Mousa A., Aboul-Fotouh A. (2009). A randomized controlled trial to assess the safety and efficacy of silymarin on symptoms, signs and biomarkers of acute hepatitis. Phytomedicine.

[59] Massodi M., Rezadoost A., Panahian M., Vojdanian M. (2013). Effects of silymarin on reducing liver aminotransferases in patients with nonalcoholic fatty liver diseases. Govaresh.

[60] Velussi M., Cernigoi A.M., De Monte A., Dapas F., Caffau C., Zilli M. (1997). Long-term (12 months) treatment with an an-ti-oxidant drug (silymarin) is effective on hyperinsulinemia, exogenous insulin need and malondialdehyde levels in cirrhotic diabetic patients. J. Hepatol..

[61] Ebrahimi-Mameghani M., Ashrafi S., Javadzadeh Y., Jafarabadi A.M. (2014). The Effect of Chlorella vulgaris Supplementation on Liver Enzymes, Serum Glucose and Lipid Profile in Patients with Non-Alcoholic FattyLiver Disease. Health Promot. Perspect..

[62] Talebi B., Jameshorani M., Salmani R., Chiti H. (2015). The effect of Chlorella vulgaris vs. Artichoke on patients with non-alcoholic fatty liver disease (NAFLD): A randomized clinical trial. J. Adv. Med. Biomed. Res..

[63] Azocar J., Diaz A. (2013). Efficacy and safety of Chlorella supplementation in adults with chronic hepatitis C virus infection. World J. Gastroenterol..

[64] Silva-Sperb A.S., Moraes H.A., Barcelos S.T.A., de Moura B.C., Longo L., Michalczuk M.T., Cerski C.T.S., Uribe-Cruz C., da Silveira T.R., Álvares-Da-Silva M.R. (2024). Probiotic supplementation for 24 weeks in patients with non-alcoholic steatohepatitis: The PROBILI81VER randomized clinical trial. Front. Nutr..

[65] Chitsaz M., Mozaffari-Khosravi H., Salman-Roghani H., Zavar-Reza J., Lotfi M. (2016). Effect of Chlorella vulgaris VS. spirulina supplementation on lipid profile and liver function in patients with nonalcoholic fatty liver disease: A randomized controlled trial. Int. J. Probiotics Prebiotics.

[66] Mazloomi S.M., Samadi M., Davarpanah H., Babajafari S., Clark C.C.T., Ghaemfar Z., Rezaiyan M., Mosallanezhad A., Shafiee M., Rostami H. (2021). The effect of *Spirulina* sauce, as a functional food, on cardiometabolic risk factors, oxidative stress biomarkers, glycemic profile, and liver enzymes in nonalcoholic fatty liver disease patients: A randomized double-blinded clinical trial. Food Sci. Nutr..

[67] Mazokopakis E.E., Papadomanolaki M.G., Fousteris A.A., Kotsiris D.A., Lampadakis I.M., Ganotakis E.S. (2014). The hepatoprotective and hypolipidemic effects of Spirulina (Arthrospira platensis) supplementation in a Cretan population with non-alcoholic fatty liver disease: A prospective pilot study. Ann. Gastroenterol..

[68] Baicuş C., Tănăsescu C. (2002). Chronic viral hepatitis, the treatment with spiruline for one month has no effect on the aminotransferases. Rom. J. Intern. Med..

[69] Ferreira-Hermosillo A., Torres-Duran P.V., Juarez-Oropeza M.A. (2010). Hepatoprotective effects of Spirulina maxima in patients with non-alcoholic fatty liver disease: A case series. J. Med. Case Rep..

[70] Aller R., Luis D., Izaola O., Conde R.M.G., Sagrado D., Primo B., La Fuente J. (2011). Effect of a probiotic on liver aminotransferases in nonalcoholic fatty liver disease patients: A double blind randomized clinical trial. Eur. Rev. Med. Pharm. Sci..

[71] Vajro P., Mandato C., Licenziati M.R., Franzese A., Vitale D.F., Lenta S., Caropreso M., Vallone G., Meli R. (2011). Effects of *Lactobacillus rhamnosus* Strain GG in Pediatric Obesity-related Liver Disease. J. Pediatr. Gastroenterol. Nutr..

[72] Javadi L., Ghavami M., Khoshbaten M., Safaiyan A., Barzegari A., Gargari B.P. (2017). The Effect of Probiotic and/or Prebiotic on Liver Function Tests in Patients with Nonalcoholic Fatty Liver Disease: A Double Blind Randomized Clinical Trial. Iran. Red Crescent Med. J..

[73] Nabavi S., Rafraf M., Somi M., Homayouni-Rad A., Asghari-Jafarabadi M. (2014). Effects of probiotic yogurt consumption on metabolic factors in individuals with nonalcoholic fatty liver disease. J. Dairy Sci..

[74] Vatsalya V., Feng W., Kong M., Hu H., Szabo G., McCullough A., Dasarathy S., Nagy L.E., Radaeva S., Barton B. (2023). The Beneficial Effects of Lactobacillus GG Therapy on Liver and Drinking Assessments in Patients with Moderate Alcohol-Associated Hepatitis. Am. J. Gastroenterol..

[75] Malaguarnera M., Vacante M., Antic T., Giordano M., Chisari G., Acquaviva R., Mastrojeni S., Malaguarnera G., Mistretta A., Volti G.L. (2012). Bifidobacterium longum with Fructo-Oligosaccharides in Patients with Non Alcoholic Steatohepatitis. Dig. Dis. Sci..

[76] Duseja A., Acharya S.K., Mehta M., Chhabra S., Rana S., Das A., Dattagupta S., Dhiman R.K., Chawla Y.K. (2019). High potency multistrain probiotic improves liver histology in non-alcoholic fatty liver disease (NAFLD): A randomised, double-blind, proof of concept study. BMJ Open Gastroenterol..

[77] Famouri F., Shariat Z., Hashemipour M., Keikha M., Kelishadi R. (2017). Effects of Probiotics on Nonalcoholic Fatty Liver Disease in Obese Children and Adolescents. J. Pediatr. Gastroenterol. Nutr..

[78] Wong V.W., Won G.L., Chim A.M., Chu W.C., Yeung D.K., Li K.C., Chan H.L. (2013). Treatment of nonalcoholic steatohepatitis with probiotics. A proof-of-concept study. Ann. Hepatol..

[79] Monem S.M.A. (2017). Probiotic Therapy in Patients with Nonalcoholic Steatohepatitis in Zagazig University Hospitals. Euroasian J. Hepato-Gastroenterol..

[80] Manzhalii E., Virchenko O., Falalyeyeva T., Beregova T., Stremmel W. (2017). Treatment efficacy of a probiotic preparation for non-alcoholic steatohepatitis: A pilot trial. J. Dig. Dis..

[81] Sharma P., Sharma B.C., Puri V., Sarin S.K. (2008). An open-label randomized controlled trial of lactulose and probiotics in the treatment of minimal hepatic encephalopathy. Eur. J. Gastroenterol. Hepatol..

[82] Gonciarz Z., Besser P., Lelek E., Gundermann K.J., Johannes K.J. (1988). Randomised placebo-controlled double blind trial on “essential” phospholipids in the treatment of fatty liver associated with diabetes. Méd. Chir. Dig..

[83] Sas E., Grinevich V., Efimov O., Shcherbina N. (2013). Beneficial influence of polyunsaturated phosphatidylcholine enhances functional liver condition and liver structure in patients with nonalcoholic steatohepatitis. Results of a prolonged randomized blinded prospective clinical study. J. Hepatol..

[84] Dajani A.I.M., Abu Hammour A.M., Zakaria M.A., Al Jaberi M.R., Nounou M.A., Semrin A.I.M. (2015). Essential phospholipids as a supportive adjunct in the management of patients with NAFLD. Arab. J. Gastroenterol..

[85] Yin D.Y., Kong L.M. (2000). Observation for curative effect of Essentiale in treatment of fatty liver caused by diabetes mellitus. Qilu Yixue Zazhi.

[86] Poongothai S., Karkuzhali K., Prakash G., Sangeetha T., Saravanan G., Deepa R., Gopalakrishnan S., Mohan V. (2005). Effect of essentiale in diabetic subjects with non-alcoholic fatty liver. Int. J. Diabetes Dev. Ctries..

[87] Arvind N., Savaikar P., Rajkumar J.S. (2006). Therapy for NAFLD—A comparative study of essential phospholipids vs ursodeoxycholic acid. Ind. J. Clin. Pract..

[88] Wu Y. (2009). Efficacy analysis of polyene phosphatidylcholine for type 2 diabetes complicated with fatty liver. Hunan Zhong Yiyao Daxue Xuebao.

[89] Li Z. (2013). Efficacy of polyene for diabetes complicated with non-alcoholic fatty liver disease. Neimenggu Zhongyiyao.

[90] Gad A.I., Elmedames M.R., Abdelhai A.R., Marei A.M., Abdel-Ghani H.A. (2021). Efficacy of vitamin D supplementation on adult patients with non-alcoholic fatty liver disease: A single-center experience. Gastroenterol. Hepatol. Bed Bench.

[91] Mohamed A.A., Halim A.A., Mohamed S., Mahmoud S.M., Eldemiry E.M.B., Mohamed R.S., Shaheen M.M., Naguib G.G., Muharram N.M., Khalil M.G. (2023). The effect of high oral loading dose of cholecalciferol in non-alcoholic fatty liver disease patients. A randomized placebo controlled trial. Front. Pharmacol..

[92] Shidfar F., Mousavi S.N., Lorvand Amiri H., Agah S., Hoseini S., Hajimiresmail S.J. (2019). Reduction of Some Atherogenic Indices in Patients with Non-Alcoholic Fatty Liver by Vitamin D and Calcium Co-Supplementation: A Double Blind Randomized Controlled Clinical Trial. Iran. J. Pharm. Res..

[93] Sakpal M., Satsangi S., Mehta M., Duseja A., Bhadada S., Das A., Dhiman R.K., Chawla Y.K. (2017). Vitamin D supplementation in patients with nonalcoholic fatty liver disease: A randomized controlled trial. JGH Open.

[94] Foroughi M., Maghsoudi Z., Ghiasvand R., Iraj B., Askari G. (2014). Effect of Vitamin D Supplementation on C reactive Protein in Patients with Nonalcoholic Fatty Liver. Int. J. Prev. Med..

