# Supplementary material for: Natural Products as Hepatoprotective Agents—A Comprehensive Review of Clinical Trials
Source: Plants (Basel). 2024 Jul 20;13(14):1985. doi: 10.3390/plants13141985 (PMC11280762; doi:10.3390/plants13141985)
Supplement: Supplementary file 1 [file plants-13-01985-s001.zip › plants-3086492-supplementary.pdf]

Supplementary material for:

Natural Products as Hepatoprotective Agents—A Comprehensive Review of Clinical Trials

Piotr Służały[1], Paweł Paśko[2] and Agnieszka Galanty[2,*]

[1] Department of Pharmacognosy, Jagiellonian University Medical College, Medyczna 9, 30-688 Cracow, Poland; piotr_s-95@o2.pl
[2] Department of Food Chemistry and Nutrition, Jagiellonian University Medical College, Medyczna 9, 30-688 Cracow, Poland; p.pasko@uj.edu.pl
* Correspondence: agnieszka.galanty@uj.edu.pl

Figure S1. Flowchart of the search strategy

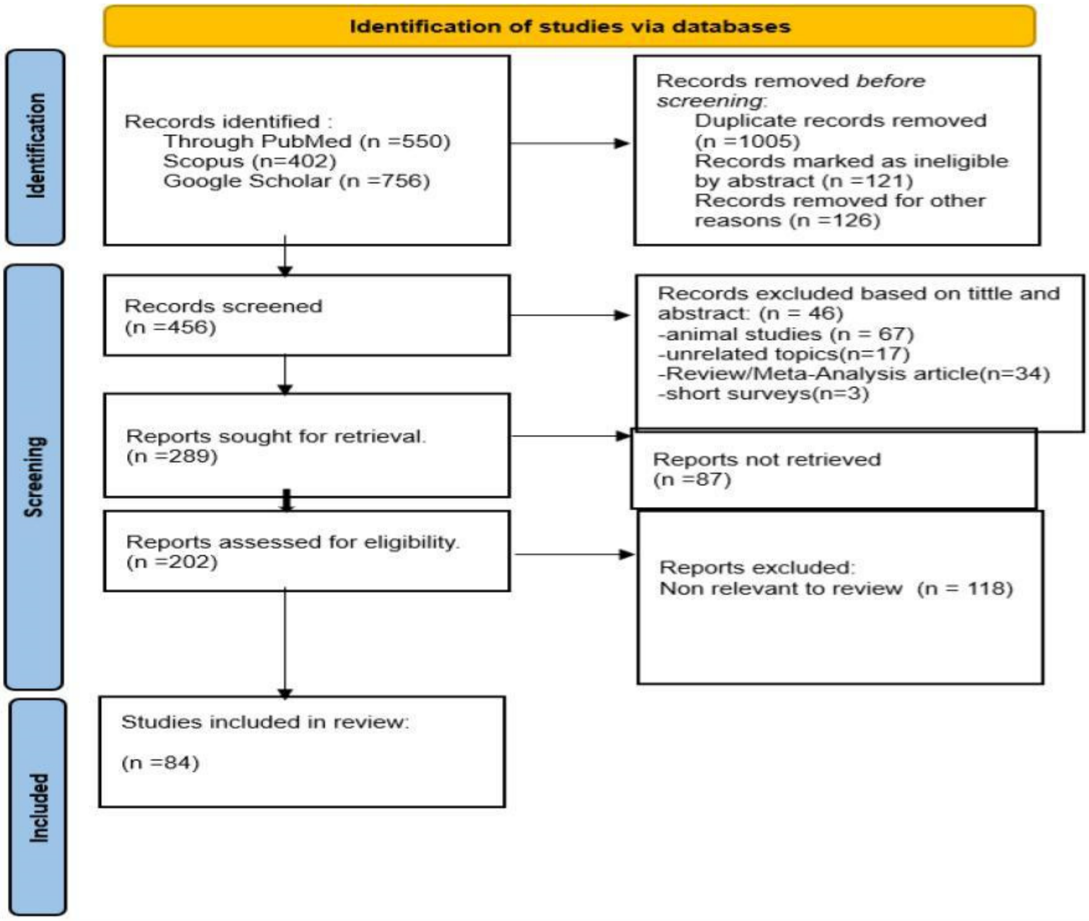

Table S1. Clinical trials of hepatoprotective effect of artichoke.

disease	artichoke form and dose	Study design	Results*	ref.
NAFLD	Artichoke leaf extract standardized on 20 mg of chlorogenic acid<br><br>400 mg/capsule	Placebo: metformin 1 g/d and vitamin E 400 IU/d, n=30 (13F, 17M)<br><br>Intervention:<br><br>1) vitamin E 400 IU/d and artichoke 800mg/d n=30 (13F, 18M)<br><br>2) metformin 1g/d and artichoke 800mg/d n=30 (10F, 20M)<br><br>for 3 months (total n=90, mean age 38)<br><br>RCT	ALT↓(-41.2%(1),-37.0%(2)/ -29.4%)<br><br>AST↓(-36.2%(1),-16.6%(2)/ -17.2%)<br><br>ALP↓(-7.8%(1),-2.5%(2)/ -1.0%)	[16]
NASH	*Cynara scolymus* in form of herb extract<br><br>450 mg/tablet	Placebo: n=30 (9F, 21M - mean age 47)<br><br>Intervention:<br><br>n=30 (9F,21M - mean age 49), 6 tablets per day (2700 mg extract)<br><br>for 2 months (total n=60)<br><br>dbRCT	ALT↓(-53%/-13.6%)<br>AST↓(-46.0%/-11.0%)	[21]
NAFLD	dried artichoke standardised to 2 mg cynarin, 200 mg/tablet	Placebo: n=40 (15F, 25M)<br><br>Intervention: n=49 (24F, 25M) 3 capsules daily (total n=89, mean age 47)<br><br>for 2 months<br><br>pilot dbRCT	ALT↓(-38%/+11.8%)<br><br>AST(-29.5%/+13.8%)<br><br>ALP↓(-2.3%/+1.5%)	[17]

NAFLD	300mg/d Bergamot Polyphenolic Fraction (BPF) and cichorium 300 mg/d extract, alone or in combination (Bergacyn; 50/50%)	Placebo: 300 mg excipients n=20<br>Intervention:<br>1) artichoke (300 mg/daily) n=20<br>2) BPF (300 mg/daily) n=20<br>3) formulation 50/50% of both extracts (300+300 mg) n=20 (total n=60, mean age 51)<br>for 4 months<br>dbRCT	ALT↓(-12%/-0.3%)<br>AST↓(-8%/-0.4%)<br>ALP↓(-15.2%/-1.2%)	[18]
hiperlipoproteinemia	artichoke dry extract. drug/extract ratio 25–35:1, aqueous extract<br>450 mg/tablet	Placebo: n=69 (23M, 46F) mean age 54.1<br>Intervention: 1800 mg/d mean age 49.7, n=72 (22M, 50F)<br>for 6 weeks<br>dbRCT	ALT↓(-15.2%/7.0-%)<br>AST↓(-17.3%/-8.3%)	[15]
moderate polygenic hypercholesterolemia	1tablet contain:dry extracts of *Cynara scolymus* and *Berberis aristata* at increased bioavailability<br>no data on single doses	Placebo: n=20 mean age: 62.6 mean weight 62.6 kg<br>Intervention: artichoke and berberis (n=20) mean age 53.8, 63.6 kg<br>for 2 months<br>dbRCT	ALT↓(-11%/-2.8%)<br>AST↓(-11.5%/-5.9%)	[19]

untreated hypercholesterolaemia	dry artichoke leaf extract (5-6 % caffeoylquinic acid) and other herbal plants[1]<br>200 mg/tablet	Placebo: placebo tablets n=15(8F, 7M)<br>Intervention:<br>1) three tablets daily n=15 (12F, 3M)<br>2) six tablets daily n=15 (11F, 4M)<br>for 4 weeks<br>dbRCT	No significant changes in liver parameters (ALT,AST)	[14]
polygenic hypercholesterolemia	dry artichoke leaf extract 200 mg (5-6 % caffeoylquinic acid and other ingredient composition[2]	Placebo: n=28 (14F, 14M) age 55<br>Intervention: n=28 (16F, 12M) age 54<br>for 8 weeks<br>dbRCT	ALT,AST,gGT↕-<br>No significant changes in liver parameters	[20]

RCT-randomized clinical trial; dbRCT- double-blind randomized clinical trial, M-male, F-female

*in reduction of liver enzymes in intervention groups/ placebo group (baseline and post intervention difference percentage)

[1]Ubiqsome® (Coenzyme Q10 phytosome) 25 mg equivalent to Coenzyme Q10 5 mg Zinc 5 mg Vazguard® (Phytosome Bergamot Polyphenolic fraction) 1000 mg Pycrinil® artichoke d.e. (Cynara cardunculus L.) 100 mg

[2]red yeast rice extract 167 mg (0.67 mg monacolin K), sugar cane extract 3.70 mg (90 % policosanols; 60 %octacosanol), dry artichoke leaf extract 200 mg (5-6 % caffeoylquinic acid), dry garlic extract 10 mg (0.8 % allicin, 1.8 % alliin), pine bark extract 6.67 mg (90 % oligomeric proanthocyanidins), vitamin E 12.86 mg, riboflavin (vitamin B2) 1.60 mg, inositol hexanicotinate (vitamin B3) 2.92 mg (equivalent to 2.67 mg nicotinic acid), dicalcium phosphate 199 mg, microcrystalline cellulose 87.36 mg, calcium citrate 63.22 mg, tricalcium phosphate 34 mg and magnesium stearate 22 mg.

Table S2. Clinical trials of hepatoprotective effect of berberine.

Study disease	berberine form and dose	Study design	Results*	ref.
NAFLD	3 tablets contain 1.5g/d of berberine extract	Placebo: n=30 (16F, 14M) age 48.7<br><br>Intervention: n=38 (21F, 17M) age 47.6,<br><br>for 3 months<br><br>dbRCT	ALT↓ (-35.2%/-6.4%)<br>AST↓ (-33.1%/-2.3%)	[26]
NAFLD	3 tablets contain: 1.5g/d of berberine extract	Placebo: n=30 received metformin<br><br>Intervention: berberine + reference of metformin standard dose n=48,<br><br>for 16 month<br><br>open-label RCT	ALT↓ (-13.8%/-3.5%)<br>AST↓(17.6-%/-6.2%)	[24]
NAFLD	berberine 0.5 g/capsule<br><br>lifestyle intervention (LSI) as a 150 minutes of exercise and diet restriction of 1500 kcal daily	Placebo: LSI n=62 (32M, 30F) ≈50.6 age<br><br>Intervention:<br><br>1) LSI + pioglitazone 15 mg/d n=60 (28M, 32F) ≈53.2 age<br><br>2) berberine 0.5g three times daily n=62 (38M, 24F) ≈50.7 age<br><br>for 16 weeks	ALT↓ (-61.8%/-24.7%)<br><br>AST↓ (-35.6%/-27.1%)<br><br>GGT↓ (-32.5%/-21%)	[23]

		open-label-RCT		
NAFLD	6.25 g of berberine daily (orally 5L water including 100g dried berberine boiled at 167F until 4L.)	Placebo: n=24 (17M, 7F) age 42.2<br><br>Intervention: n=24 (20M, 10F) age 40.6<br><br>for 7 weeks<br><br>open label-RCT	ALT↓(-20.1%/-3.2%)<br><br>AST↓(-25.9%/-6.2%)	[22]
NASH	berberine (as ursodeoxycholate, an ionic salt of berberine) 500 mg/ tablet	Placebo: n=33 (11M, 22F)<br><br>Intervention:<br><br>1) 1000 mg/day berberine n=33(7M, 26F)<br><br>2) 2000 mg/day berberine n=34 (10M, 24F)<br><br>for 18 weeks<br><br>dbRCT	ALT↓(-8.7%/-3%)(1)/ (- 19%/-3%)(2)<br><br>GGT↓(-29.69%/-2%)(1)/ (- 30%/-2%)(2)	[25]

RCT-randomized clinical trial; dbRCT- double-blind randomized clinical trial, M-male, F-female *in reduction of liver enzymes in intervention groups/ placebo group (baseline and post intervention difference percentage)

Table S3. Clinical trials of hepatoprotective effect of chicory.

**Study disease**	**Form and dose of chicory**	**Study design**	**Results***	**ref.**

NAFLD	200 ml of chicory plant extract daily plus aerobic training	Placebo: obese boys in age 13-15 received a similar extract in color n=12,<br><br>Intervention: obese boys in age 13-15 n=12, 100 mL in the morning and at night<br><br>for 8 weeks<br><br>RCT	ALT↓(-18.3%/-13.7%)<br><br>AST↓(-11.3%/-7.4%)	[34]
NAFLD	brewed chicory leaf prepared from 15 g/d of freely available chicory leaves	Placebo: n=30 for both group adult individuals with grade 2 or 3 NAFLD<br><br>Intervention: n=26 received chicory 15g/d<br><br>for 6 weeks<br><br>dbRCT	ALT↓(-26.1%/+2.0%)<br><br>AST↓(-14.2%/+3.4%)	[35]
overweight patients with NAFLD	9g/d of powdered chicory 96 seeds (4.5 g infused for 30 min in 100 mL hot water two times a day between meals).	Placebo: received similar tablets n=21 (7M, 14F) age 40.38<br><br>Intervention:<br><br>1) 9 g/d of powdered chicory n=21(11M, 10F) age 42.57<br><br>2) 3 g/d turmeric n=21 (12M, 9F) age 42.67<br><br>3) combination of 2)+3) n=22 (8M, 14F) age 41.59<br><br>for 12 weeks<br><br>dbRCT	ALT↓(-13.9%(1),-21.1%(2),-17.50%(3)/-3.50%)	[31]
NAFLD	200 ml of chicory plant extract plus aerobic training	Placebo: women n=10 received placebo solution<br><br>Intervention: women n=10 no specified age took chicory extract<br><br>for 8 weeks<br><br>RCT	ALT↓ (-18.4%/-7.3%)<br><br>AST↓(-19.6%/-13.6%)	[29]

cirrhotic patients	one tablet liv-52 contain *Cichorium intybus* 65 mg_and others[1]	Placebo: n=18 (16M, 2F) age 46.39 received identical tablets in shape and color<br>Intervention: n=18 (16M, 2F) age 52.7 received three tablets daily of liv-52<br>for 6 months<br>dbRCT	ALT↓(-56.7%/-33.0%)<br>AST↓(-35.9%/-22.4%)	[32]
NAFLD	8 ml (400 mg of the herbal extract) of herbal syrup before eating breakfast and also at bedtime	Placebo: n=27 no sex specified. age of patients 18-65<br>Intervention: chicory 8 ml twice daily n=28, no sex specified,<br>for 8 weeks<br>RCT	ALT↓(-52.2%/-0.7%)<br>AST↓(-46.6%/-2.8%)<br>ALP↓(-24.5%/-1.3%)	[27]
NAFLD	500 mg/day of powder extract of root of *C. intybus*	Placebo n=30 (24M, 6F) age 41.8 BMI 30.5<br>Intervention with chicory n=31 (27M, 4F) age 41.2 BMI 31.0<br>All subjects advised to take a low-fat, low carbohydrate diet, to do regular sport activities; and to lose more than 4 kg. for 8 weeks<br>RCT	ALT↨(-19.7%/-21.0%)<br>AST↨(-13.7%/-25.1%)	[30]
hepatobiliary disease	cichory and *Solanum nigrum* as a fresh herb juice of 100ml twice daily	Intervention: fresh herb juice, n=30 (17M, 13F) age 25<br>for 5 weeks<br>non placebo, prospective, single-arm,single-blinded pre and post analytical clinical study	ALT↓(-78.2%)<br>AST↓(-69.0%)<br>ALP↓(-24.2%)	[28]
NAFLD	*Cichorium intybus* L. and *Cinnamon* mixture infusion (2.5 and 0.5 g/100 mL and twice/day)	non control group<br>Intervention: infusion twice daily, n=25 (11M, 14F)<br>for 4 weeks<br>before-after clinical trial	ALT↓(-29.7%)<br>AST↓(-59.7%)<br>ALP↓(-4.7%)	[33]

RCT-randomized clinical trial; dbRCT- double-blind randomized clinical trial, M-male, F-female *in reduction of liver enzymes in intervention groups/ placebo group (baseline and post intervention difference percentage)[1]contain *Capparis spinosa* 65 mg, *Cichorium intybus* 65 mg, *Solanum nigrum* 32 mg, *Cassia occidentalis* 16 mg, *T. arjuna* 32 mg, *A. millefolium* 16 mg, *Tamarix gallica* 16 mg. It also contains 'Mandur bhasma' (33 mg/tablet) which is prepared from ferric oxide

Table S4. Clinical trials of hepatoprotective effect of turmeric/curcumin.

Study disease	form and dose of curcumin/turmeric	Study design	Results*	ref.
NAFLD	One tablet contain 800 mg phytosomal curcumin (Curserin®1 200 mg curcumin)	Placebo: n=40 (21F, 19M) age 53<br>Intervention: two tablets daily of curcumin n=40 (22F, 18M) age 54.3<br>for 8 weeks<br>dbRCT	ALT↓(-34.8%/-12.5%)<br>AST↓(-23.1%/-8.7%)<br>GGT↓(-14.3%/-3.7%)	[40]
NAFLD	Turmeric (2 g/daily) as oral capsules	Placebo: n=32 (19M, 13F) age 38.56<br>Intervention: n=32 (19M, 13F) age 44.12<br>for 8 weeks<br>dbRCT	ALT↓(-22.9%/+11.5%)<br>AST↓(-21.0%/-7.4%)<br>GGT↓(-24.2%/-4.0%)	[44]
NAFLD	The sinacurcumin® dose 80 mg/day (two 40-mg capsules per day)	Placebo: two identical non-curcumin capsules n=42 (23M, 19F) age 42.5<br>Intervention: two capsules of nanocurcumin 80 mg/d n=42 (23M, 19F) age 41.8<br>for 3 months<br>RCT	ALT↓(-23.8%/-5.9%)<br>AST↓(-22.5%/-7.1%)	[41]
Liver cirrhosis	oral dose of 1000 mg curcumin per day divided into two equal doses of one 500 mg capsule (curcumin, Karen Pharmaceutical, Iran)	Placebo: similar capsules n=31(17M, 14F)<br>Intervention: 1000 mg/day curcumin n=29 (14M, 15F)<br>for 3 months<br>dbRCT	ALT↓(-11.2%/-7.0%)<br>AST↓(-11.7%/+16.9%)<br>ALP↓(-16.7%/-2.2%)	[37]

NAFLD	Curcumin was administered in the form of 500 mg capsules(Meriva®; Indena S.p.A)	Placebo: group n=43 (27M, 16F) age 47.21<br><br>Intervention: curcumin 1000 mg/day in two divided doses n=44 (22M, 20F) age 44.98<br><br>for 8 weeks<br><br>RCT	ALT↓(-29.9%/+12.2%)<br><br>AST↓(-25.2%/+13.8%)	[43]
NAFLD	500 mg curcuminoids co-administered with 5 mg piperine daily (curcumin capsules C3 Complex, Sami Labs Ltd, Bangalore, India)	Placebo: similar tablets n=35 (16M, 19F) age 47.51<br><br>Intervention: 500 mg curcuminoids and 5 mg piperine daily n=35 (20M, 15F) age 46.63<br><br>for 12 weeks<br><br>dbRCT	ALT↓(-25.1%%/-3.3%)<br>AST↓(-27.6%/-17.9%)<br>ALK↓(-22.6%/-0.1%)	[42]
NAFLD	amorphous dispersion curcumin formulation (500 mg/day equivalent to 70 mg curcumin)	Placebo: the same capsules in color and shape, n=40 (19M, 21F) age 48.95<br><br>Intervention: 500 mg/d curcumin n=40 (19M, 21F) age 46.37<br><br>for 8 weeks<br><br>RCT	ALT↓(-7.7%/-5.3%)<br>AST↓(-17.5%/+6.3%)	[39]
NAFLD	Each capsule contains 500 mg of curcumin per day. Curcumin capsules were filled withBCM-95 (BIO-CURCUMIN®) , a proprietary combination of 95% curcuminoids and essential oil of turmeric-ar-turmerone.	Placebo: identical capsules n=23(14M, 9F) age 45.13<br><br>Intervention: three capsules/daily n=27 (13M, 14F) age 46.19<br><br>both groups were encourage to introduce lifestyle modification for 12 weeks<br><br>RCT	ALT↨(-5.6%/-6.8%)<br>AST↨(-2.7%/-3.5%) GGT↨(-1.6%/1.7%)	[38]

RCT-randomized clinical trial; dbRCT- double-blind randomized clinical trial, M-male, F-female *in reduction of liver enzymes in intervention groups/ placebo group (baseline and post intervention difference percentage)

[1]120 mg phosphatidylserine, 480 mg phosphatidylcholine and 8 mg piperine from *Piper nigrum* L. dry extract)

Table S5. Clinical trials of hepatoprotective effect of green tea.

Study disease	form and dose of green tea	Study design	Results*	ref.
NASH	600 mg of green tea tablets with catechins per day(epigallocatechin gallate 52.6%)	Placebo: placebo tablets n=12 (9M,3F)<br>Intervention: green tea n=26 (13M,13F)<br>for 24 weeks<br>dbRCT	ALT↓(-4.2%/+39.6%)<br>AST↓(-6.1%/+75.7%)<br>γ-GT↓(-7.1%/+32.1%)	[46]
NAFLD in obese patients	capsule of green tea extract 500mg: epigallocatechin gallate (EGCG) 31.43% weight	Placebo: n=40 (28M, 12F) age 28 BMI 28.6<br>Intervention: 500 mg of green tea extract n=40 (26M, 14F) mean age 25, BMI 29.5<br>for 12 weeks<br>RCT	ALT↓(-25.0%/-2.7%)<br>AST↓(-32.7%/+5.5%)	[48]
NAFLD with obese patients	one capsule containing 500 mg of either Green Tea Extract (GTE) or cellulose each day (31.43% weight of EGCG)	Placebo: cellulose tablet each day<br>Intervention: green tea extract (GTE) supplement (500 mg GTE tablet per day) n=35 (16M, 19F) age: 20-50<br>For both groups lifestyle modifications were advised<br>for 12 weeks<br>dbRCT	ALT↓(-34.2%/-17.4%)<br>AST↓(-39.5%/-25.9%)<br>ALP↓(-62.8%/-7.0%)	[45]
NAFLD	Green tea adjusted to 1,080 mg/ 700 ml catechin content.	Placebo: green tea-flavoured beverage (0 mg/700 ml catechin content) n=5, age 54.2<br>Intervention: green tea n=7, mean age 47.1<br>for 12 weeks	ALT↓(-42.1%/-3.1%)	[49]

		dbRCT		
NAFLD	Each tablet contain 550mg of green tea catechin	Placebo: starch tablet daily, n=24 age 39.5<br>Intervention: one tablet daily, n=21, mean age 41<br>for 12 weeks<br>dbRCT	ALT↓(-37.6%/-11.2%)<br>AST↓(-26.7%/-0.9%)	[47]

RCT-randomized clinical trial; dbRCT- double-blind randomized clinical trial, M-male, F-female

*in reduction of liver enzymes in intervention groups/ placebo group (baseline and post intervention difference percentage)

Table S6. Clinical trials of hepatoprotective effect of schisandra.

**Study disease**	**form and dose of schisandra**	**Study design**	**Results***	**ref.**
mild liver injury	one capsule contain 7.5 mg of Fructus schizandra fruit extract capsules<br>placebo herbal combination[1]	Placebo: extract[1] that has used water as a solvent n=30(17M, 13F)<br>Intervention: six capsules of schisandra fructus n=30 (20M, 10F)<br>for 6 weeks<br>RCT	ALT↓(-53.5%/-64.1%)<br>AST↓(-59.2%/-58.0%)	[51]
borderline liver dysfuncti-on	one tablet contain Schisandrin B 0.06–0.12 mg and sesamin 2.25–2.95 mg	Placebo: four placebo tablets with starch n=20 (10M, 10F) mean age 51.8<br>Intervention: four tablets a day of schisandra +sesamin n=20 (10M, 10F) mean age 46.2<br>for 5 months<br>dbRCT	ALT↓(-41.8%/+3.6%)<br>AST↓(-25.6%/+2.1%)	[52]

RCT-randomized clinical trial; dbRCT- double-blind randomized clinical trial, M-male, F-female

*in reduction of liver enzymes in intervention groups/ placebo group (baseline and post intervention difference percentage)

[1](combination of Curcuma longa rhizomes, Curcuma xanthorrhiza rhizomes, and Taraxacum officinale leaves), 50 mg of aerosil, 50 mg of lactose, 100 mg of Avicel PH102, and 8 mg of stearate)

Table S7. Clinical trials of hepatoprotective effect of milk thistle/silymarin

Study disease	form and dose of silymarin	study design	Results*	ref.
NAFLD	each tablet contain silybum marianum extract (540.3 mg) plus vitamin E (36 mg)	Placebo: similar tablets and advise to change lifestyle n=18 (11M, 7F)<br>Intervention: two tablets daily and lifestyle modification n=18 (11M, 7F)<br>for 3 months<br>RCT	ALT ↕(-6.6%/-22.7%)<br>AST↕(-2.8%/-13.5%)<br>GGT↓(-43.3%/-37.5%)	[57]
Acute hepatitis (viral aetiology)	Each capsule of silymarin contained 140 mg silymarin as active ingredient	Placebo: low-dose multivitamin and mineral compound three times daily- it meet daily allowance, n=50 (44M, 6F) mean age 29<br>Intervention: three capsules daily n=55 (42M, 13F) mean age 31<br>for 8 weeks<br>RCT	ALT,AST↕ no significant changes	[58]
Hepatitis C virus (HCV)	each capsule contain dry extract of milk thistle fruits 140 mg, marketed as Legalon 140®	Placebo group n=52 (41M, 11F) mean age 56 BMI 29.1<br>Intervention:<br>1) group A received 420 mg of silymarin n=50 (34M, 16F) mean age 54 BMI 28.5<br>2) group B received 720 mg of silymarin n=52 (35M, 17F) age 54 BMI 30.2<br>for 24 weeks<br>RCT	ALT↓(-10.3%(1),-13.8%(2)/-4.1%)	[55]

chronic hepatitis	600 mg silymarin - of *Silybum marianum* extract containing 80% silymarin/tablet	Placebo: identical tablet n=8<br>Intervention:<br>1) group I received 600 mg of silymarin n=8<br>2) group II received 1200 mg of silymarin n=8<br>all n=24 mean age 43<br>for 12 weeks<br>RCT	ALT,AST↕ no significant changes	[54]
NAFLD	Livergol tablets containing 140 mg of silymarin active extract	Placebo: identical starch tablets twice daily n=50 (29M, 21F) mean age 39.0 BMI 27.80<br>Intervention: two tablets daily of Livergol n=50 (28M, 22F) mean age 39.28 BMI 26.75<br>for 6 months<br>RCT	ALT↓(-35.6/-14.0%)<br>AST↓(-30.5/-9.4%)	[53]
NASH	140 mg of silymarin/tablet	Placebo: identical tablets with starch n=50 (31M, 19F) mean age 48.32 BMI 29.18<br>Intervention: one tablet n=50 (31M, 19F) mean age 48.42 BMI 29.04<br>for 3 months<br>RCT	ALT↓(-18.5%/-1.2%)<br>AST↓(-24.0%/-2.2%)	[59]
Liver disease no specified	one tablet contain silymarin (Legalon® 420 mg/day)	Placebo: identical tablets n=50 (54M, 6F) mean age 38.8<br>Intervention: one tablet n=47 (39M, 8F) mean age 35.2<br>for 4 weeks<br>dbRCT	ALT↓(-62.2%/-16.1%)<br>AST↓(-57.5%/-30.7%)	[56]
Cirrhotic (diabetic) patients	each capsule contain 200 mg of silymarin (Legalon®, Italy)	Placebo: identical capsules in shape and colour, n=30 mean age 62 BMI 24.9<br>Intervention: 600 mg silymarin n=30 mean age 63 BMI 25.1<br>for 12 months<br>dbRCT	ALT↓(-24.2%/-4.3%)<br>AST↓(-11.8%/+4.5%)	[60]

RCT-randomized clinical trial; dbRCT- double-blind randomized clinical trial, M-male, F-female

*in reduction of liver enzymes in intervention groups/ placebo group (baseline and post intervention difference percentage)

Table S8. Clinical trials of hepatoprotective effect of chlorella

**Study disease**	**form and dose of chlorella**	**study design**	**Results***	**ref.**
chronically infected with hepatitis C virus	dry pulverized *Chlorella pyrenoidosa* powder plus a water soluble extract of 500 mg in capsule	patients unable to get antiviral treatment for HCV, treated with six tablets of chlorella<br>n=13, age or sex no specified<br>for 12 weeks<br>non-placebo cohort study	ALT ↓(-18.6%)<br>AST↓ (-19.9%)	[64]
NAFLD	chlorella in form of powder tablets 500 mg each	Placebo: similar two tablets as intervention, n=66 sex and age non specified<br>Intervention: two tablets with chlorella<br>for 8 weeks<br>RCT	ALT↓(-7.2%/+2.7%)	[65]
NAFLD	300 mg tablets of *C. vulgaris* (commercially available under the name of ALGOMED®[1]	Placebo: vitamin E 400 mg/d n=29 (15M, 14F)<br>Intervention: chlorella 1.2 g+ vitamin E 400 mg/d n=26 (15M, 9F)<br>for 8 weeks<br>dbRCT	ALT↓(-30.3%/-13.4%)<br>AST↓(-37.1%/-27.8%)<br>ALP↓(-15.8%/-1.4%)	[61]
NAFLD	300 mg tablets of *C.vulgaris*[1]	Placebo: metformin 1.25 g/d+ Vit.E 200 mg/d n=43, age 47.1<br>Intervention: chlorella 1.2 g/d + Vit. E 200 mg/d n=33, age 51<br>for 12 weeks<br>open-label RCT	ALT↕ (-10.9%/-81.0%)<br>AST↓(-14.9%/+28.7%)<br>ALP↕(+47.5%/-42.6%)	[62]

NAFLD	chlorella in form of powder tablets 500mg	Placebo: metformin 1.25 g/d+ Vit.E 200 mg/d n=30, age 51<br>Intervention: chlorella 1.5 g/d + Vit. E 200 mg/d n=31, age 48<br>for 8 weeks<br>open label RCT	ALT↓ (-13.3%/+3.2%)<br>AST↓(-8.4%/+2.1%)	[63]

RCT-randomized clinical trial; dbRCT- double-blind randomized clinical trial, M-male, F-female

*in reduction of liver enzymes in intervention groups/ placebo group (baseline and post intervention difference percentage) 1(Bioprodukte Prof.steinberg Produktions- und Vertriebs GmbH & Co KG,Germany)

Table S9. Clinical trials of hepatoprotective effect of spirulina.

Study disease	form and dose of spirulina	study design	Results*	ref.
Chronic viral hepatitis	each tablet contain 400 mg of spirulina	Placebo: eight identical tablets n=12, mean age 45<br>Intervention: eight tablets of spirulina n=12 mean age 49<br>for 4 weeks<br>dbRCT	no significant changes in liver parameters (ALT, AST↕)	[69]
NAFLD	spirulina in 500 mg tablets	Placebo: two identical tablets n=20, mean age 42<br>Intervention: 2 tablets daily n=20 mean age 42.8<br>for 8 weeks<br>RCT	ALT↓(-7.2%/+2.7%)	[65]
NAFLD	4.5 g/day *Spirulina maxima* in tablet form (0.5 g each).	non placebo group n=3 male 43.77 age and female age 44<br>for 3 months<br>case report	ALT↓(-43.7%)	[70]
NAFLD	one sachet of sauce containing 2 g of spirulina daily	Placebo: identical sachet with placebo n=23 (13M,10F) mean age 35.78 BMI 25.41<br>Intervention: one sachet of spirulina n=23 (9M, 14F) mean age 38.87 BMI 24.67<br>for 8 weeks	ALT↓(-13.6%/-1.5%)<br>AST↓(-17.1%/-1.2%)<br>ALP↓(-6.3%/-2.1%)	[66]

		dbRCT		
NAFLD	spirulina (two sachets of 3 g daily)	non placebo group, no label and open trial n=15 (13M, 2F) mean age 47.9 BMI 32.7 for 6 months<br>non-randomized trial	ALT↓(-37.5%)<br>AST↓(-38.5%)<br>yGT↓(-26.7%)	[67]

RCT-randomized clinical trial; dbRCT- double-blind randomized clinical trial, M-male, F-female

*in reduction of liver enzymes in intervention groups/ placebo group (baseline and post intervention difference percentage)

Table S10. Clinical trials of hepatoprotective effects of probiotics.

**Study disease**	**form of administration, number and strain of bacteria**	**study design**	**Results***	**ref.**
NAFLD	One tablet contain 500 million of: *L. bulgaricus* and *S. thermophilus*	Placebo: tablet 120 mg of starch n=14 (10M, 4F) age 44.3 BMI 29.5<br>intervention: probiotic tablet n=14 (10M, 4F) age 49.4 BMI 30.2<br>for 3 months<br>dbRCT	ALT↓(-10.8/+6.8%)<br>AST↓(-13.8%/+14.8%)<br>yGT↓(-8.9%/+1.8%)	[70]

NAFLD	Each capsule containing 112.5 billion live, lyophilised, lactic acid bacteria and bifidobacteria, namely *L. paracasei* DSM 24733, *L. plantarum* DSM 24730, *L. acidophilus* DSM 24735 and *L.delbrueckii* subsp. *bulgaricus* DSM 24734, *B. longum* DSM 24736, *B. infantis* DSM 24737, B. breve DSM 24732, and *S. thermophilus* DSM 24731,[1]	Placebo: two capsules three times daily with starch n=20 (15M, 5F) age 33<br>Intervention: advised to take two capsule three times a day (675 billion bacteria) n=19 (13M, 6F) age 38 for 12 months<br>dbRCT	ALT↓(-55.4%/-35.5%) AST↓(-47.1%/-39.5%)<br>ALP↓(-25.9%/-12.3%)	[71]
NAFLD in children	One capsule containing *L. acidophilus* ATCC B3208, 3 109 colony forming units [CFU]; *B. lactis* DSMZ 32269, 6 109 CFU; *B. bifidum* ATCC SD6576, 2 109 CFU; *L. rhamnosus* DSMZ 21690, 2 109 CFU)	Placebo: identical capsule n=32 (18M, 14F) age 12.6 BMI 26.61<br>Intervention: one capsule daily n=32 (14M, 18F) age 12.7 BMI 26.44<br>for 12 weeks<br>triple-blinded RCT	ALT↓(-29.6%/-9.3%)<br>AST↓(-24.5%/-11.9%)	[77]

NAFLD	2 billions of *B. longum* and *L. acidophilus* (probiotic) or inulin 10g/d(prebiotic)	Placebo: n=19 (13M, 6F) age 42.21 BMI 30.38<br>Intervention:<br>1) probiotic group n=20 (17M, 3F) age 43.9 BMI 29.91<br>2) Prebiotic n=19 (16M, 3F) age 38.68 BMI 30.96<br>3)Prebiotic(FOS)+probiotic n=17 (14M, 3F) age 43.24 BMI 32.30<br>for 3 months<br>dbRCT	ALT↓(-21.2%(1),-16.4%(2), -21.1%(3)/+0.4%)<br>AST↓(-32.1%(1),-21.2%(2)-28.0%(3)/-4.5%)<br>ALP↓(-9.7%(1),-0.4%(2),-6.6%(3)/-1.2%)<br>GGT↓(-10.1%(1),-10.4%(2),-16.8%(3)/+3.2%)	[73]
NASH	*B. longum* and Fos (+2.5 g, vitamin B1 (1.4 mg), vitamin B2 (1.6 mg), vitamin B6 (2.0 mg), and vitamin B12 (1.0 mg). LSI-Lifestyle Intervention	Placebo: identical capsule in shape, colour +LSI n=34 (15M, 17F) age 46.7<br>Intervention: *B. longum* +FOS n=34 (18M, 16F) age 46.9 +LSI<br>for 24 weeks<br>dbRCT	ALT↓(-53.4%/-39.5%)<br>AST↓(-63.9%/-42.9%)	[75]
NASH	containing *L. casei*, *L. rhamnosus*, *L. bulgaris*, *B. longum*, and *S. thermophilus* (10bilion bacteria /capsule in total)	Placebo: n=37(16M, 21F) age 43.5 BMI 26.6<br>Intervention: probiotic capsule n=38(11M, 27F) age 44.3 BMI 26.4<br>for 12 weeks<br>open-label RCT	ALT↓(-31.3%/-3.8%)<br>AST↓(-24.1%/+13.6%)<br>GGT↓(-21.6%/-18.3%)	[80]

NASH	Acidophilus capsule (*L. acidophilus*, which contains 2 billion viable organism, and mixture of rice flour, gelatin, and magnesium stearate)	Placebo: three identical capsules, n=15 (8M, 7F) age 44.33 BMI 33.05<br>Intervention: capsules was given to patients 30 minutes before meal three times daily, n=15 (9M, 6F) age 44.2 BMI 32.56<br>for 1 month<br>RCT	ALT↓(-44.7/-1.2%)<br>AST↓(-33.1%/-4.9%)	[79]
NAFLD	*L. acidophilus* La5 and *B. lactis* Bb12 on d 1 were 6.46 × 106 and 4.97×106 cfu/g, respectively. Probiotic yoghurt contained 2.39 × 106 cfu/g of *L. acidophilus* La5 and 2.08 × 106 cfu/g of B. lactis Bb12	Placebo of conventional yoghourt n=36 (18M, 18F) mean age 44.05<br>Intervention: probiotic yoghurt (300 g daily) n=36 (17M, 19F) mean age 42.75<br>for 8 weeks<br>dbRCT	ALT↓(-19.0%/+3.9%)<br>AST↓(-15.4%/+3.9%	[74]
Minimal hepatic encephalo pathy	Each capsule containing *S. faecalis* 60 million, *C. butyricum* 4 million, *B. mesentricus* 2 million, lactic acid bacillus 100 million	Placebo: 30-60ml of lactulose daily n=35 age 39.5<br>Intervention:<br>1) probiotic group received three capsules daily n=35 age 43.5<br>2) lactulose+probiotic n=35 age 43.7, for sex ratio male:female=3.63<br>for 1 month<br>RCT	ALT↓(-31.0%(1), - 27.8%(2)/-20.0%<br>AST↓(- 38.4%(1),-27.2%(2)/- 35.8%	[81]
obesity-related liver disease in children	oral *L. rhamnosus* (12 billion CFU/day)	Placebo: n=10 (9M, 1F), mean age of both group 10.7<br>Intervention: probiotic n=10 (9M, 1F)<br>for 8 weeks<br>dbRCT	ALT↓(-43.0%/-3.1%)	[72]

NASH	Each 10 g sachet 200 million probiotic cultures contained *L. plantarum*, *L. deslbrueckii*, *L. acidophilus*, *L. rhamnosus* and *B. bifidum*. Other ingredients of the formula included 3 g of fructo oligosaccharides (prebiotics), cellulose, magnesium stearate, silica and milk.	Placebo: identical sachet without probiotics n=10 (5M, 5F) mean age 55<br>Intervention: one sachet twice daily for 6 months n=10 (8M, 2F) mean age 42<br>for 6 months<br>RCT	ALT↓(-44.1%/+2.8%)<br>AST↓(-26%/+60.5%)	[78]

RCT-randomized clinical trial; dbRCT- double-blind randomized clinical trial, M-male, F-female

*in reduction of liver enzymes in intervention groups/ placebo group (baseline and post intervention difference percentage) 1sold in Europe, Singapore, USA and Korea under the brand VIVOMIXX, VISBIOME and DESIMONE FORMULATION

Table S11. Clinical trials of hepatoprotective effect of phospholipids.

**Study disease**	**form and dose of phospholipids**	**study design**	**Results***	**ref.**
NAFLD + diabetes or obesity	1800mg/d divided in three capsules of EPL	non placebo group, intervention group for n=40, no specified sex or age,<br>for 3 months<br>open-label RCT	ALT↓(-12.4%)	[87]
NAFLD	1800 mg of EPL a day was given for 24 weeks, followed by 900 mg for 48 weeks.	non placebo group, n=113 (62M, 51F) age 43.5 BMI 29.3<br>for 48 weeks<br>randomized open label study	ALT↓(-52.87%)<br>AST↓(-31.94%)	[84]

histologically proven fatty liver disease	One capsule of Essentiale forte contained 300 mg of essential phospholipids (EPL) tocopherol and vitamins of the B group.	Placebo: identical six capsules n=15 (8M, 7F)<br>Intervention: six capsules daily in three doses, N=15 (4M, 11F)<br>for 6 months<br>RCT	GGT↓(-21.2%/-12.5%)	[82]
NAFLD + diabetes	1800 mg/d divided in six capsules of EPL	non placebo group, n=86 (57M, 29F) mean age 51,<br>for 6 weeks<br>open-label RCT	ALT↓(-3.8%)	[89]
NAFLD + diabetes type 2	1800 mg/d divided in three capsules of EPL	non placebo group, n=22 (11M, 11F) mean age 41 BMI 28.2,<br>for 6 months<br>dbRCT	ALT↓(-17.4%)<br>AST↓(-10.4%)<br>GGT↓(-22.6%)	[86]
NASH+ diabetes	1800 mg/d divided in three capsules of EPL	no placebo trial n=189<br>for 6 months<br>prospective randomized open-label study	ALT↓(-21.4%)<br>AST↓(-12.5%)	[83]
NAFLD + diabetic	1800 mg/d divided in three capsules of EPL	no placebo group, n=100 (64M, 36F) mean age 55,<br>for 1 months<br>RCT	ALT↓-12.03%	[88]

NAFLD + diabetes	1800 mg/d divided in three capsules of EPL	Placebo: similar capsules metformin 1.5g/d n=62<br>Intervention: EPL and metformin 1.5 g/d n=63<br>total number n=125 (73M, 52F) age 59<br>for 3 months<br>open-label RCT	ALT↓(-15.36%/-2.42%)	[85]

RCT-randomized clinical trial; dbRCT- double-blind randomized clinical trial, M-male, F-female

*in reduction of liver enzymes in intervention groups/ placebo group (baseline and post intervention difference percentage)

Table S12. Clinical trials of hepatoprotective effect of vitamin D.

**Study disease**	**form and dose of vitamin D**	**participants of trials**	**Results***	**ref.**
NAFLD	capsule containing 50 000 IU Vitamin D	Placebo: one identical capsules weekly as intervention n=30 (14M, 16F) BMI 32.4 age 48.5<br>Intervention: one capsule of vit. D once weekly n=30 (15M, 15F) BMI 31.2 age 48.5<br>for 10 weeks<br>RCT	ALT↓(-7.5%/-2.1%)<br>AST↓(-17.0%/-9.3%)	[94]
NAFLD	single intramuscular dose of 200 000 IU cholecalciferol(D3)	Placebo: IM injection (normal saline 0.9%) n=40 (13M, 27F) mean age 46 BMI 29.8<br>Intervention: IM injection once monthly n=40 (13M, 27F) mean age 47 BMI 30.6<br>for 6 months<br>single-blind, non-randomized trial	ALT↓(-16.3%/+4.8%)<br>AST↓(-19.0%/+5.6%)	[90]

NAFLD	single oral dose of cholecalciferol 200 000 IU followed by oral cholecalciferol 800 IU.<br>the standard conventional therapy: regular exercise in the form of any physical activity in addition to calorie restriction	Placebo: standard conventional therapy in addition to placebo n=70 (35M, 25F) mean age 54<br>Intervention: standard conventional therapy n=70 (31M, 29F) mean age 52<br>for 4 months<br>dbRCT	ALT,AST↕ no significant changes	[91]
NAFLD	a single injection of vitamin D (600 000 IU) given intramuscularly	Placebo: saline injection and introduce lifestyle changes n=30 (19M, 11F) mean age 40 BMI 27.0<br>Intervention: vitamin d injection and introduce lifestyle modification n=51 (36M, 15F) mean age 37 BMI 28.0<br>for 6 months<br>RCT	ALT↓(-32.2%/-3.1%)	[93]
NAFLD	one tablet containing 1000 IU of vitamin D (25 µg/d as calciferol)	Placebo: n=36 (23M, 13F) age 44.1<br>Intervention:<br>1) vitamin D n=37 (22M, 15F) mean age 39.81<br>2) vitamin D + 500 mg calcium carbonate n=37 (23M, 14F) age 38.3<br>for 12 weeks<br>dbRCT	ALT↓(-7.6%(1),<br>-9.6%(2)/+1.1%)<br>AST↓(-13.8%(1),-17%(2)/ -8.0%)	[92]

RCT-randomized clinical trial; dbRCT- double-blind randomized clinical trial, M-male, F-female

*in reduction of liver enzymes in intervention groups/ placebo group (baseline and post intervention difference percentage)
